# Structural Characterization of Four Redox States of the P‑cluster in Molybdenum Nitrogenase via Electrochemical Control of Crystals

**DOI:** 10.1021/jacs.6c08522

**Published:** 2026-08-26

**Authors:** Shoba Laxmi, William K. Myers, Zhi-Yong Yang, Lance C. Seefeldt, Stephen B. Carr, Kylie A. Vincent

**Affiliations:** † Department of Chemistry, University of Oxford, Inorganic Chemistry Laboratory, South Parks Road, Oxford OX1 3QR, U.K.; ‡ Research Complex at Harwell, Rutherford Appleton Laboratory, Harwell Campus, Didcot OX11 0QX, U.K.; § Centre for Advanced Electron Spin Resonance (CAESR), 6396 University of Oxford, Oxford OX1 3QR, U.K.; ∥ Department of Chemistry and Biochemistry, 4606 Utah State University, Logan, Utah 84322, United States

## Abstract

We report X-ray crystallographic structures of the *Azotobacter vinelandii* nitrogenase MoFe protein showing the 8Fe-7S electron-transfer P-cluster in four redox states. Using electrochemical poising of protein crystals, together with *in crystallo* EPR spectroscopic verification of the redox state, we obtain structures showing the P-cluster at P^N^, P^1+^, P^2+^, and P^3+^ levels. This provides a detailed structural characterization of P-cluster rearrangement between the catalytically relevant P^N^ and P^1+^ levels and the first experimental confirmation that the *S* = 7/2 P^3+^ state is structurally similar to P^2+^. These studies pave the way for future understanding of the structure–function relationship in nitrogenase catalysis.

## Graphical abstract

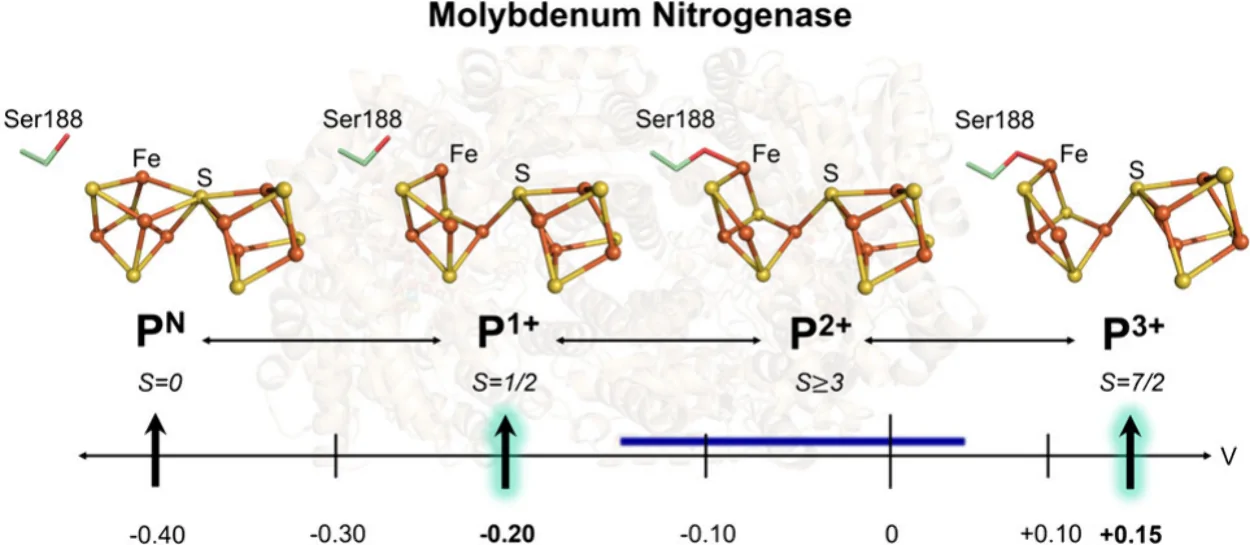

The substantial energy cost of N_2_ fixation via the high temperature/pressure Haber-Bosch process brings urgency to the search for new routes to ammonia. Electrochemical N_2_ reduction is one alternative for localized ammonia generation for fertilizers or as a renewable, carbon-free fuel for the shipping industry.^1–5^ Akin to electrochemical N_2_ reduction, biological N_2_ fixation involves accumulation of multiple electrons and protons at the catalytic site. Within molybdenum iron (MoFe) nitrogenase, this is the Mo-7Fe-9S-C-homocitrate active site cluster, termed ‘FeMoco’ or ‘M-cluster’. Electrons are delivered to FeMoco sequentially from the 8Fe-7S ‘P-cluster’ (Figure 1A). In turn, these electrons are supplied from the partner ‘Fe protein’, in a process linked intimately to ATP-hydrolysis.^6^ Timing of electron delivery is likely a critical aspect of the catalytic mechanism. There is still uncertainty about how the P-cluster delivers electrons to an ever-more reduced active site, at biologically relevant potentials.

**Figure 1:**
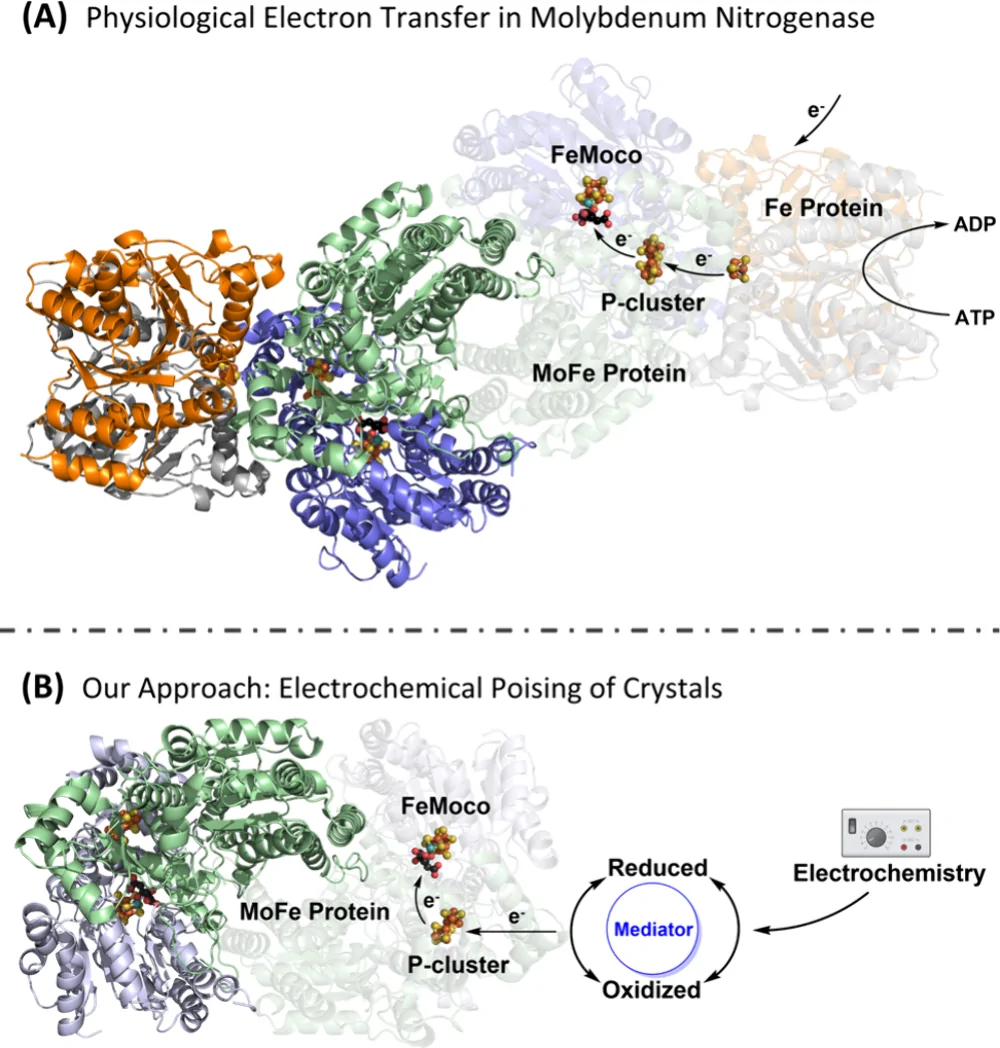
Schematic representation of (A) native electron transfer in nitrogenase (PDB: 1N2C) and (B) electrochemical poising of nitrogenase MoFe protein crystals. MoFe protein comprises α (blue) and β (green) subunits as an α_2_β_2_ tetramer.

Protein-bound iron–sulfur clusters are typically rigid, to minimize reorganization energy for fast electron transfer, and are generally involved in single-electron transfer steps.^7–9^ A notable exception is the proximal 4Fe-3S cluster of O_2_-tolerant NiFe hydrogenases, where structural change provides access to an additional redox level, allowing exchange of an extra electron.^10,11^ Multielectron catalysis at the active site of nitrogenase is also thought to involve cluster rearrangement,^12–14^ and additionally, metal-bound hydrides aid storage of the multiple electrons needed for N_2_ reduction.^15–17^

Structural changes have also been observed in the nitrogenase P-cluster. Three redox states, P^N^ ([8Fe^II^-7S]), P^1+^ ([7Fe^II^Fe^III^-7S]), and P^2+^ ([6Fe^II^2Fe^III^-7S]) are thought to be physiologically relevant.^6,18–20^ At pH 8, midpoint potentials *E*(P^1+/^P^N^) and *E*(P^2+^/P^1+^) are both reported as −309 mV but separate at lower pH, likely due to protonation of specific states.^21^ Other more oxidized states (P^3+^, P^superox^) are reported (*E*(P^3+/^P^2+^) = +90 mV at pH 8, ESI Scheme S1).^21,22^

The P cluster can be considered as two fused cubanes, labeled “proximal” and “distal”, based on their proximity to FeMoco (ESI Figure S1). In P^N^, the distal portion adopts a [4Fe-4S] cubic conformation which breaks up in the P^2+^ state with **Fe6** instead coordinated by the side-chain of a conserved serine (**β-Ser** ^**188**^), and **Fe5** by the backbone amide of cysteine (**α-Cys** ^**88**^).^23,24^

The complexity of electron delivery to MoFe protein under turnover conditions has hindered the characterization of redox-dependent changes in nitrogenase clusters. Structures revealing P^2+^ and P^N^ were achieved using chemical oxidants or reductants,^23^ but intermediate P^1+^ has not been characterized in pure form.^25^ Alternative ways of supplying electrons to MoFe protein in solution include photoactivated electron transfer from CdS nanocrystals,^26^ and mediated transfer from electrodes.^27–29^ Exploiting mediated electron transfer, here we apply an electrochemical poising methodology to *crystals* of *Azotobacter vinelandii* nitrogenase MoFe protein, building on an approach we previously demonstrated for hydrogenases.^30,31^ Solvent channels in MoFe protein crystals have diameter ≥10 Å. This is large enough to allow dissolved organic mediators to access the P-cluster from the protein surface for mediated electron transfer (Figure S2). This allows us to navigate reversibly between near-homogeneous populations of redox states within crystals (Figure 1B). Here, we isolate crystals predominantly (P^1+^) or entirely (P^N^, P^2+^, P^3+^) in distinct redox states, as verified by EPR spectroscopy (Figure 2) on slurries of microcrystals (50 μm – 70 μm, Figure S3). Electrochemically poised crystals retained their diffraction quality, enabling us to obtain complete structures for each poised crystal.

**Figure 2:**
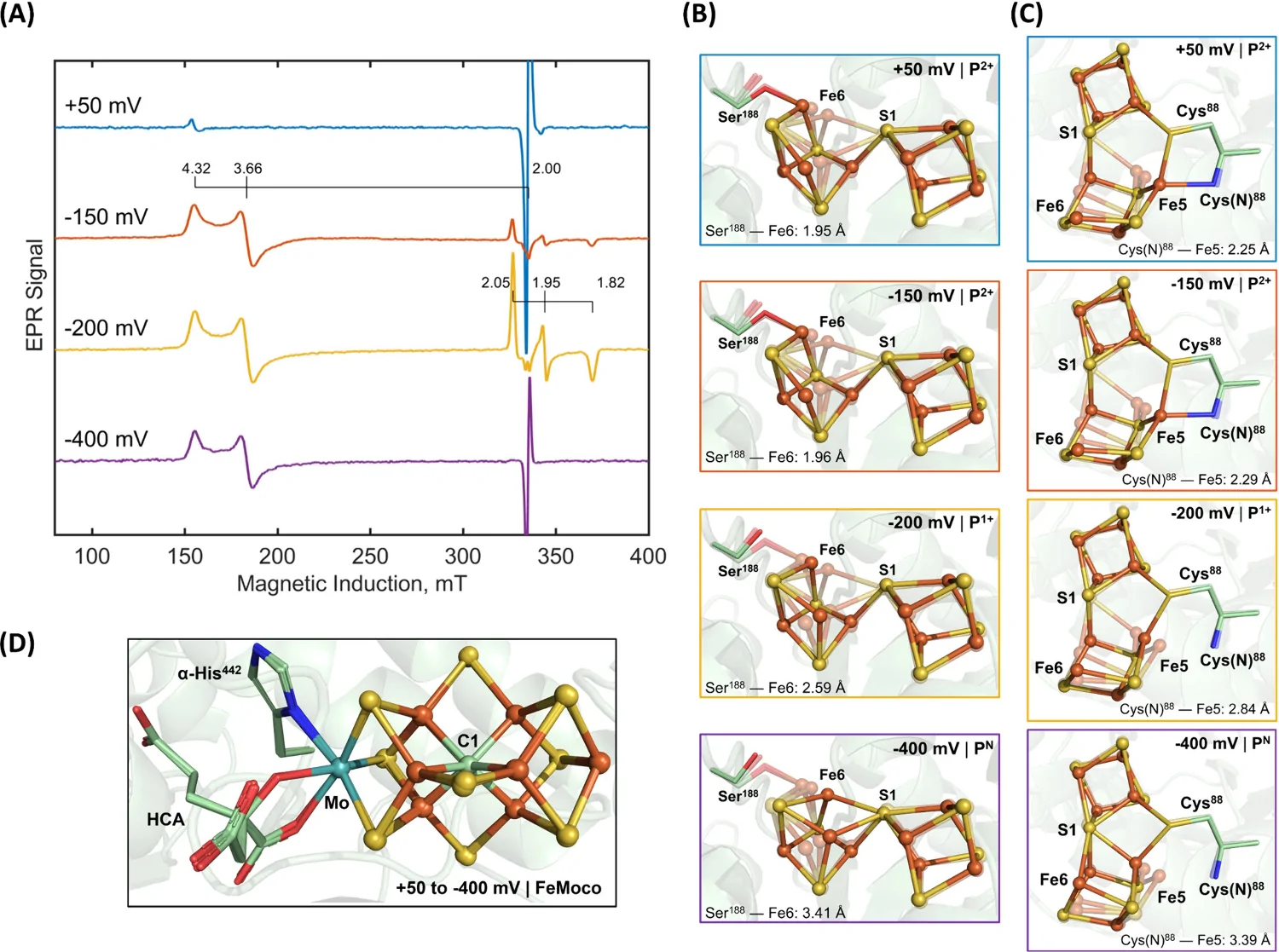
(A) CW-EPR, X-band perpendicular mode for MoFe protein microcrystals as a function of potential. Changes around the P-cluster as it cycles through P^2+^, P^1+^ and P^N^ states with potential, viewed with respect to (B) β-Ser^188^, (C) α-Cys^88^. (D) Superimposed structures of FeMoco. (Atom labels: red = oxygen, orange = iron, yellow = sulfur, green = carbon, blue = nitrogen and teal = molybdenum.) Electron density maps associated with each poised structure are shown in Figures S9–S11 and difference maps in Figure S12.

The EPR spectrum of microcrystals poised at −400 mV, (Figure 2A) displays the resting state (M^N^) of the FeMoco signal (*S* = 3/2, *g*_⊥_ = 4.32, 3.66) and an organic radical signal from reduced methyl viologen (*g*_⊥_ = 2.0, Figure S5), which obscures the *g*_⊥_ = 2.0 signal from FeMoco (Figure S6). Corresponding parallel mode EPR (Figure S7) shows no discernible signals, consistent with the P-cluster being in the all-ferrous, EPR-silent (*S* = 0), P^N^ state.^23^ X-ray diffraction data collected from larger crystals (100–300 μm) poised at the same potential (Figure 2B) show the P-cluster adopting the established “closed” conformation of the P^N^ state.^23,32^ The central sulfur atom (**S1**) is octahedrally coordinated by three iron atoms from the distal and proximal halves of the cluster, respectively. Each iron is tetrahedrally coordinated by two further bridging sulfides and one cysteine. The structure of FeMoco (Figure 2D) is identical to established resting state (M^N^) structures.^32^

Crystals poised at −200 mV show EPR features at *g*_⊥_ = 2.05, 1.95 and 1.82, consistent with P^1+^ (Figure 2A and Figure S8),^21,20^ and M^N^ (*S* = 3/2). No signal attributable to P^2+^ (*g*_∥_ = 11.9) was observed (Figure S7). The structure for crystals poised at −200 mV shows a distinct change in the distal half of the P-cluster (Figure 2). Two iron atoms, **Fe5** and **Fe6**, now assume an ‘intermediate’ conformation relative to established structures of P^N^ and P^2+^,^23^ while the proximal half remains unchanged. The positions of **Fe5** and **Fe6** were confirmed by calculation of iron anomalous difference maps (Figure S11). After refinement, residual mFo-DFc density consistent with a trace amount of the EPR silent P^N^ state was observed at the P-cluster. Comparing residual peaks with calculated omit maps suggests ∼10% of the cluster remains in the P^N^ state, while 90% of the cluster is P^1+^ (Table S3)—a considerably higher proportion of P^1+^ than had been isolated previously (*vide infra*).

In the P^1+^ state, substantial elongation of the Fe–S bond distances was observed for the two iron atoms that have moved upon oxidation of the cluster (Table S4). Average bond distances **S1-Fe6** and **S1-Fe5** are 3.34 and 3.01 Å, respectively, at −200 mV, suggesting they are no longer coordinating (Fe–S bond lengths are between 2.20–2.50 Å).^8^ Temperature factors of the iron atoms in P^1+^ are also elevated relative to the rest of the cluster and P^N^/P^2+^ states, implying metastability.

At −150 mV, perpendicular mode EPR of poised crystals show a significant lowering of signal intensity corresponding to P^1+^ (ESI Section S7.3), while the *S* = 3/2 resting state of FeMoco is still observed (Figure 2A). However, a prominent *g* = 11.9 peak in parallel mode EPR, indicative of the *S* ≥ 3 P^2+^ state appears (Figure S7).^22,33,34^ The relative populations estimated by EPR serve only as a comparative assessment of spectral changes, because accurate spin quantification is not possible for microcrystal slurries using current EPR sample preparations. The solved structure shows the P-cluster adopting an ‘open’ conformation (Figure 2B,C) consistent with P^2+^ assignment. Here, **Fe5** and **Fe6** have moved further from **S1**. **Fe5** becomes coordinated by the amide of **α-Cys** ^**88**^ and **Fe6** is coordinated by the oxygen of **β-Ser** ^**188**^. No residual electron density corresponding to P^1+^ was observed postrefinement.

A potential of +50 mV was then applied. As at −150 mV, an absorption-shaped peak matching reported values for P^2+^ in chemically oxidized solution samples^18,22,35^ was detected (*g* = 11.9, Figure S7). In perpendicular mode (Figure 2A), the *S* = 3/2 feature was absent, indicating oxidation of FeMoco to the M^ox^ state (*E*(M^ox/^M^N^) = −40 mV);^36^ further features were observed, characteristic of adventitious Fe^3+^ (*g* = 4.3)^22,37^ and an intense, mediator radical signal (*g*_⊥_ = 2.0, Figure S5). The corresponding structure exhibits the ‘open’ conformation expected for P^2+^ (Figures 2B,C), matching that of −150 mV (Figure S12). Notably, FeMoco at the M^ox^ level remains structurally indistinguishable from the M^N^ cluster seen at negative potentials (Figure 2D). This is consistent with published structures (Figure S10).

Across the four structures discussed above (+50, −150, −200 and −400 mV), there were negligible structural changes in the protein backbone (root-mean square deviation for Cα = 0.07–0.10 Å, Figure S13). Transitions between the P^2+^, P^1+^, and P^N^ redox states were accompanied by minimal conformational changes in amino acid residues, both along the pathway between the P-cluster and FeMoco, and at the Fe protein docking interface. However, previous studies have shown that Fe protein docking and ATP hydrolysis induce conformational dynamics that propagate throughout the MoFe protein.^38–40^ Looking ahead, it will be interesting to determine whether electrochemical poising can be extended to crystals of the MoFe–Fe protein complex, and how the P-cluster redox state influences protein dynamics within this assembly.

The electrochemically poised structures for P^N^ (−400 mV) and P^2+^ (−150 and +50 mV) agree with published structures for the reduced and doubly oxidized P-cluster (Tables S9 and S11).^41–44,23,45^ Moving from P^2+^ to P^1+^ to P^N^ the **Ser** ^**188**^ **-Fe6** and the **Cys­(N)** ^**88**^ **-Fe5** distances increase to become noncoordinating in P^1+^ and P^N^ states (Table S4). This is broadly consistent with earlier QM/MM calculations by Ryde and co-workers.^24^ **Ser** ^**188**^ and **Cys­(N)** ^**88**^ have been suggested as protonation sites.^21^ Although protonation would not be detectable in the X-ray diffraction data, redox-linked protonation may play a role in the decoordination of these residues from **Fe6** and **Fe5** in the reduced P-cluster.

An earlier attempt to structurally characterize the P^1+^ state of MoFe protein suggested a “half-open” conformation but was hampered by crystals that showed extreme radiation sensitivity, resulting in an incomplete (60%) data set.^25^ The P^1+^ structure was assigned, using peaks in the mFo-DFc maps to infer a different position of metals Fe5 and Fe6 from either the P^N^ or P^2+^ state. However, any conclusions drawn from this model must be treated with caution given the low completeness of the data.^25^ Subsequent QM/MM analysis was consistent with these crystals containing a mixture of states (P^1+^ and P^2+^).^24^ For VFe protein of V nitrogenase, a 1.20 Å structure reported by Einsle and co-workers also showed evidence of P^1+^ as a half-open conformation, however this state only represented 40% of a mixture with P^N^ (Figure S14).^14^

Knowledge of oxidized P-cluster states beyond P^2+^ is limited. Although P^3+^ is not considered relevant to nitrogenase catalysis, access to an expanded redox manifold for an iron–sulfur framework should be of interest in bioinorganic chemistry. Earlier EPR redox titrations revealed P^3+^ as a mixed spin system—rhombic *S* = 1/2 (*g* = 1.91, 1.88, 1.68), coupled with an axial *S* = 7/2 with three thermally isolated low field Kramers doublets, *g*_z,eff_ (14, 10.5, and 5.5).^22,37^ Here, we show crystals poised at +150 mV resemble a clean P^3+^ *S* = 7/2 system (broad feature at *g*_eff_ = 14 in both modes, Figure 3A and Figures S15–S17). The absence of a P^2+^ signal (*g* = 11.9) indicates full conversion to P^3+^,^22^ while lack of *S* = 3/2 suggests that FeMoco remains as M^ox^. This allowed us to structurally characterize the P^3+^ state for the first time (Figure 3B,C). The P-cluster at the P^3+^ level adopts an ‘open’ conformation, with coordination of **Ser** ^**188**^ and **Cys­(N)** ^**88**^ resembling that in P^2+^ (Table S13). This agrees with earlier QM/MM calculations that modeled P^3+^ as structurally similar to P^2+^.^24^ Superposition of the higher electron density region from the +150 mV structure with the +50 mV structure shows negligible structural distortion in FeMoco (Figure 3D). Current mechanistic descriptions of nitrogenase turnover generally involve only the P^N^, P^+^, and P^2+^ states, with cluster breakdown inferred at the P^3+^ level.^20,46^ Intriguingly, our structure of the P^3+^ state shows no evidence of breakdown.

**Figure 3:**
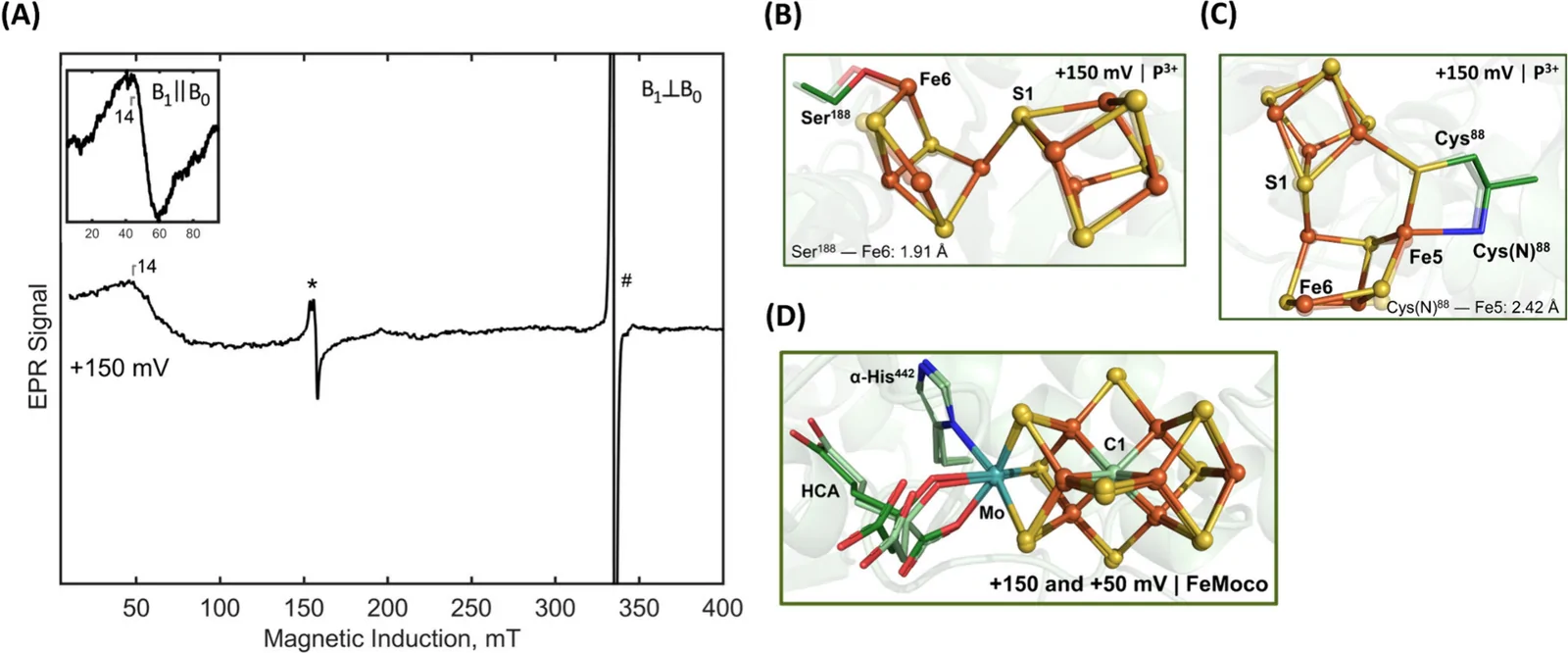
(A) X-band EPR at 10 K of MoFe microcrystals at +150 mV in perpendicular mode and parallel mode (inset). Perpendicular mode EPR collected at 9.3792 GHz with 10 mW microwave power and 0.5 mT field modulation. Features at *g* = 4.3 (*) and 2.0 (#) are attributed to a rhombic, high-spin Fe^3+^ and poised mediator (Figure S5). Parallel mode EPR collected at 9.3717 GHz with 100 mW microwave power and 0.5 mT field modulation. Changes around the P-cluster at +150 mV (dark green) and +50 mV with respect to (A) β-Ser^188^, (B) α-Cys^88^ and (D) FeMoco region.

By combining electrochemical poising of MoFe protein crystals with EPR spectroscopy and X-ray crystallography, we have trapped, and structurally characterized, discrete FeMoco (M-cluster) and P-cluster oxidation states within the MoFe protein, as summarized in Table 1. Importantly, this enabled us to determine the X-ray structure of MoFe protein in the near homogeneous (90%) P^1+^ state, and notably, the mild electrochemical poising methodology allowed collection of a complete X-ray diffraction data set. This state is a crucial intermediate between P^2+^ and P^N^, where sequential structural changes presumably contribute to the cluster’s ability to exchange two sequential electrons at very similar potentials under certain conditions. The P^1+^ structure reveals a ‘half-open’ conformation with **Ser** ^**188**^ and **Cys­(N)** ^**88**^ uncoordinated to **Fe6** and **Fe5** similar to P^N^, although at shorter distances. This raises an intriguing possibility of a transient “serine-on” configuration at the P^1+^ redox level, potentially associated with a very negative potential that facilitates delivery of low-potential electrons to FeMoco during the most demanding steps of N_2_ reduction. In the future, it would be particularly interesting to examine redox-linked structure change in crystals of the complex between MoFe and Fe protein. Additionally, the first structure for the P^3+^ state is revealed. The possibility of obtaining precise potential control in nitrogenase crystals paves the way for future work to address how cluster rearrangement enables delivery of electrons between the redox centers of nitrogenase during catalysis.

**Table 1:** Summary of Electrochemically Poised States in MoFe Protein Crystals, pH 6

Potential (mV)	–400	–200	–150	+50	+150
P-cluster	P^N^ (*S* = 0)	P^1+^ (*S* = $\frac{1}{2}$ )	P^2+^ (*S* ≥3)	P^2+^ (*S* ≥3)	P^3+^ (*S* = $\frac{7}{2}$ )
FeMoco	M^N^ (*S* = $\frac{3}{2}$ )	M^N^ (*S* = 3/2)	M^N^ (*S* = 3/2)	M^ox^ (*S* = 0)	M^ox^ (*S* = 0)

## Supplementary Material



## Notes

Description of experimental protocols including MoFe protein production, crystallization, X-ray data collection and structure determination, electrochemical manipulation, EPR spectroscopy measurements. Figures of P-cluster nomenclature, solvent channels, crystals and microcrystals of MoFe protein, voltammetry of mediators, additional EPR measurements at different potentials, 2mFo-DFc, mFo-DFo, and iron anomalous maps for each potential, asymmetric unit of poised MoFe protein structures, comparison of P-cluster structures. Scheme of midpoint potentials of P-cluster redox states. Tables of crystallographic refinements, population percentage of P^1+^, and comparison of bond lengths and angles of P-cluster structures in the redox states (PDF)

## Notes

PDB: 29UL - MoFe P^3+^ (+150 mV), 29UP - MoFe P^2+^ (+50 mV), 29UM - MoFe P^2+^ (−150 mV), 29UN - MoFe P^1+^ (−200 mV) and 29UO - MoFe P^N^ (−400 mV), and associated structure factors described in this work can be obtained for free of charge via worldwide Protein Data Bank (wwPDB).
