# Supplementary material for: Structural Characterization of Four Redox States of the P‑cluster in Molybdenum Nitrogenase via Electrochemical Control of Crystals
Source: J Am Chem Soc. 2026 Aug 26;148(35):37583–8. doi: 10.1021/jacs.6c08522 (PMC13564416; doi:10.1021/jacs.6c08522)
Supplement: Supplementary file 1 [file ja6c08522_si_001.pdf]

Supporting Information

# Structural Characterization of Four Redox States of the P-cluster in Molybdenum Nitrogenase via Electrochemical Control of Crystals

Shoba Laxmi [a, b], William K. Myers [a, c], Zhi-Yong Yang [d], Lance C. Seefeldt [d, *], Stephen B. Carr [a, b, *], Kylie A. Vincent [a, *]

[a] Department of Chemistry, University of Oxford, Inorganic Chemistry Laboratory, South Parks Road, Oxford, UK

[b] Research Complex at Harwell, Rutherford Appleton Laboratory, Harwell Campus, Didcot, OX11 0QX, UK

[c] Centre for Advanced Electron Spin Resonance (CAESR), University of Oxford, Oxford OX1 3QR, UK

[d] Department of Chemistry and Biochemistry, Utah State University, Logan, Utah 84322, USA

**S1. General Methods 3**

S1.1 Protein Production and Crystallization ... 3

S1.2 Electrochemistry ... 3

S1.3 Poising of MoFe Crystals ... 4

S1.4 EPR Spectroscopy ... 4

S1.5 X-Ray Data Collection and Structure Determination ... 5

**S2. P-Cluster Nomenclature 6**

**S3. Solvent Channels in MoFe Crystals 7**

**S4. X-ray data collection and refinement statistics 8**

**S5. Images of MoFe protein crystals and microcrystals 9**

**S6. Cyclic Voltammetry and $E_{1/2}$ of Mediators 10**

**S7. Additional EPR Spectral data 11**

S7.1 Buffer Control Spectra ... 11

S7.2 Resting State MoFe Protein Microcrystal Spectrum ... 12

S7.3 Estimation of Population using Electron Density Maps and EPR simulations of relative signal intensity of $P^{1+}$ ... 13

S7.4 Parallel Mode EPR Spectra ... 14

S7.5 Perpendicular Mode, Low-Field EPR Spectra ... 15

**S8. Additional Structural Data 16**

S8.1 2mFo-DFc Maps Around the P-Cluster ... 16

S8.2 2mFo-DFc Maps Around the M-Cluster ... 17

S8.3 Comparison of P-Cluster and local environment between redox states ... 18

Chain E ... 18

Chain A ... 19

Chain C .......... 20
Chain G .......... 21
**S9. Comparison between published P-Cluster Redox States of MoFe protein and this work 22**
S9.2 $P^N$ .......... 22
S9.2 $P^{1+}$ .......... 23
S9.3 Is the P-cluster of *M. infernus* MoFe protein also in the $P^{1+}$ state? .......... 23
S9.4 $P^{2+}$ .......... 24
**S10. Calculated Iron Anomalous Maps 25**
**S11. Calculated Difference Maps 26**
**S12. Root Mean Square Deviation of Poised Structures 27**
**S13. Comparing $P^{1+}$ state in MoFe and VFe proteins 28**
**S14. $P^{3+}$ State Characterization 30**
S14.1 Variable Temperature Parallel Mode EPR Spectra .......... 30
S14.2 Variable Temperature Perpendicular Mode EPR Spectra .......... 31
S14.3 Thermal Population .......... 32
S14.4 Comparing $P^{3+}$ and $P^{2+}$ state in MoFe protein .......... 33
S14.5 Electron Density (2mFo-DFc) maps around P-cluster .......... 34
**S15. References 35**

## S1. General Methods

### S1.1 Protein Production and Crystallization

MoFe protein from *A. vinelandii* was expressed and purified as previously described[1,2]. Purified MoFe protein was stored in 50 mM tris pH 8 buffer containing, 200 mM NaCl, 5 mM biotin, 2 mM sodium dithionite. For crystallization experiments, MoFe protein was diluted to a concentration of 20 mg/mL, using 50 mM tris pH 8 buffer containing 200 mM NaCl. Crystallization was performed in a $N_2$-filled glovebox (<1 ppm $O_2$) at ambient temperature (~23 °C).

Crystals for X-ray diffraction measurements were grown using the sitting drop vapor diffusion method, with 1.5 µL protein mixed with an equal volume of crystallization buffer (0.1 M sodium citrate pH 5.0, 0.16 M ammonium acetate, 17% (v/v) PEG Smear High, 25% (v/v) glycerol) and equilibrated against 500 µL crystallization buffer. Brown, diamond-shaped and rod-shaped crystals appeared after 2-3 days achieving maximum dimensions after 1 week. Over the course of crystal growth, it is assumed that dithionite would have been quenched. Two different protein preparations were used in this work, accounting for the difference in crystal resolution between the +150 mV structure and other structures determined in this study.

Microcrystal slurries for EPR measurements were grown in a modified vapor diffusion experiment as follows: protein volume was increase to 9 µL and mixed with 6 µL of crystallization buffer and a further 3 µL of crystal seeds (in crystallization buffer containing 20% PEG Smear High). The enlarged drops were again equilibrated against 500 µL of crystallization buffer. A crystal seed stock was made by crushing single MoFe crystals using a glass "seed bead" kit (Hampton Research, USA) following the manufacturers protocol. The resulting 50 µL seed stock was diluted to a final volume of 150 µL before addition to the crystallization experiments. Microcrystals appeared after 12 hours incubation at 23 °C.

### S1.2 Electrochemistry

Unless otherwise stated, all electrochemical experiments were carried out in MoFe cryogenic crystallization buffer, "cryo-buffer", (0.1 M sodium citrate pH 5.0, 0.16 M ammonium acetate, 20% PEG Smear High, 20% glycerol). All electrochemical manipulations were performed using a PalmSens4 potentiostat in cryo-buffer within a $N_2$-filled glovebox (<1 ppm $O_2$), at 22 °C (ambient temperature of glovebox). Organic mediators: flavin mononucleotide, methylene blue, methyl viologen, phenosafranin and phenazine methosulfate were used as purchased from Sigma-Aldrich, Merck.

Cyclic voltammograms were recorded with a standard three electrode set-up in MoFe cryo-buffer using a glassy carbon working electrode, Pt wire counter electrode (Thermo Scientific, 99.95%, 12 cm × 0.5 mm) in a 0.5 M NaCl-filled fritted tube (6 mm OD) and saturated calomel electrode (SCE) reference (Palmsens, UK). Ferrocenemethanol was used as an external standard to calibrate the reference

electrode. All potentials are quoted against the standard hydrogen electrode (SHE) using the correction $E_{SHE} = E_{SCE} + 241$ mV, at 25 °C.[3]

## S1.3 Poising of MoFe Crystals

A three-electrode bulk electrolysis setup comprising a saturated calomel reference (PalmSens, UK), a carbon felt working electrode (Thermo Scientific, 99% pure, 70 mm × 31.8 mm × 30 mm), and a Pt wire counter electrode (Thermo Scientific, 99.95%, 120 mm × 0.5 mm) in a 0.5 M NaCl-filled fritted tube (6 mm OD), was employed for poising of mediators at the following potentials: +150 mV (phenazine methosulfate), +50 mV (methylene blue), +150 mV (flavin mononucleotide), -200 mV (phenosafranin) and -400 mV (methyl viologen). Bulk electrolysis was conducted on the mediators until there was minimal change to the measured current, indicating the poised mediator had equilibrated with the electrode potential.

The open circuit potential of the cryo-buffer containing poised mediator was measured before, during and after the experiment to ensure that the crystals were subjected to the same potential for the duration of the experiment prior to flash cooling. The pH of the poised cryo-buffer was measured to be pH 6. The protocol for poising single crystals and microcrystals followed a previously published procedure.[4]

## S1.4 EPR Spectroscopy

The X-band CW-EPR was acquired in the Centre for Advanced ESR (CAESR) in the Department of Chemistry, University of Oxford. The spectrometer was an EMXmicro with a Premium bridge with Bruker BioSpin resonators ER4122SHQE-W1 for perpendicular mode and ER4116DM resonator for parallel mode. The sample temperature was controlled with an Oxford Instruments ITC-503S and ESR900 cryostat with cryogenic flow from a liquid helium dewar. Q-band tubes were loaded into an in-house customized X-band tube holder under liquid nitrogen before loading into the cryostat. Data were normalized to spectrometer settings by Bruker's Xenon software, and to temperature, Q-factor and field modulation by post-processing. Non-saturating conditions were determined in microwave power and slow scan rates.

Perpendicular CW EPR of poised microcrystal slurries was obtained by subtracting buffer & mediator control sample (giving rise to inverted mediator signals). Unless otherwise stated, measurements were performed at 12 K, microwave power 80 uW, modulation amplitude 0.9 mT, with 4-8 sweeps of 300 seconds, averaged. Microwave frequencies were 9.3935(5) GHz for +50, -200, and -400 mV samples, and 9.3794(1) GHz for the -150 mV sample (Figure 2A, main text).

## S1.5 X-Ray Data Collection and Structure Determination

After poising, crystals were flash cooled in liquid $N_2$ prior to collection of X-ray diffraction data. To minimize the possibility of radiation damage, diffraction data were collected in dose-dependent mode at beamline I04 and I03 (Diamond Light Source, UK) using X-rays of wavelength 0.95 Å and 0.97 Å (+150 mV) with an Eiger2 XE 16M detector (Table S1). Diffraction images were collected at 100 K with an oscillation angle of 0.10° over 360° rotation. Data reduction was performed using DIALS[5] and AIMLESS[6] within the Xia2[7] pipeline. Initial phase estimates were generated by molecular replacement in Phaser[8] using PDB entry 6UG0 as a search model. The resulting maps were inspected in COOT[9] followed by iterative rounds of refinement in REFMAC5[10] and manual rebuilding (COOT) until no further improvements were possible. This was followed by final rounds of refinement, including TLS refinement of temperature factors, in Phenix.[11] All figures were made in PyMOL.[12]

The proportion of different states of the P-cluster was estimated by deleting Fe atoms from the proximal half of the cluster, calculating omit maps and comparing peak heights in the resultant mFo-DFc map with any uninterpreted peaks that persisted after structure refinement.

Crystals poised at +50 mV, -150 mV, -200 mV and -400 mV displayed orthorhombic pseudosymmetry with unit cell parameters close to that expected for spacegroup $P2_12_12_1$, however structure refinement revealed the true spacegroup to be $P2_1$ with a beta angle of almost exactly 90 degrees (Table S1). The quality of the resulting models was assessed using Molprobity.[13]

We note the +150 mV poised single crystal was solved in the spacegroup *C*2 and crystals diffracted to lower resolution (2.5 Å), with poorer density on one half of the $\alpha_2\beta_2$ tetramer. This is not a result of electrochemical poising but due to inherent crystal quality for crystals grown from a different protein batch.

## S2. P-Cluster Nomenclature

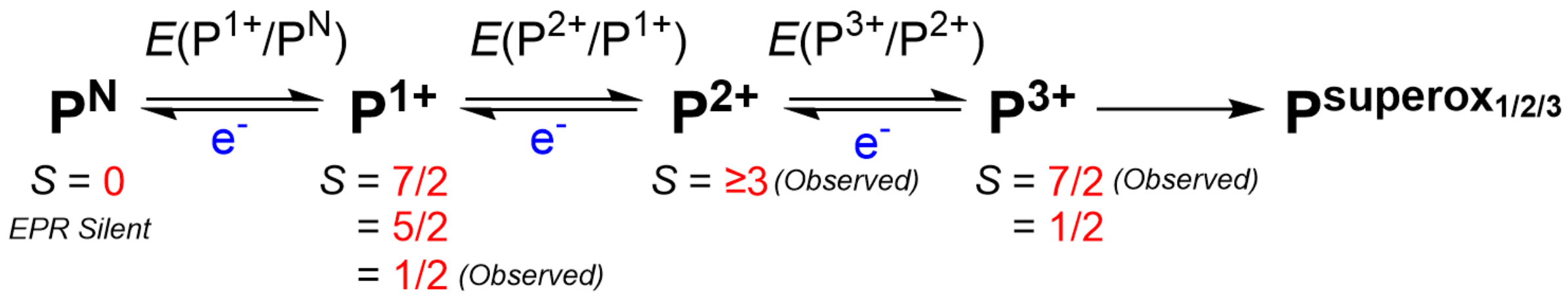


Reported values, pH 8:

$E(P^{1+}/P^{N}) = E(P^{2+}/P^{1+}) = -309$ mV

$E(P^{3+}/P^{2+}) = +90$ mV

**Scheme S1.** MoFe protein P-cluster redox states with associated midpoint potentials reported at pH 8 for the one electron couples[14] and reported spin state(s) ($S$) observed under various conditions[15]. Spin states for the electrochemically poised microcrystal redox states observed in this work, at pH 6, are indicated as '*Observed*'.

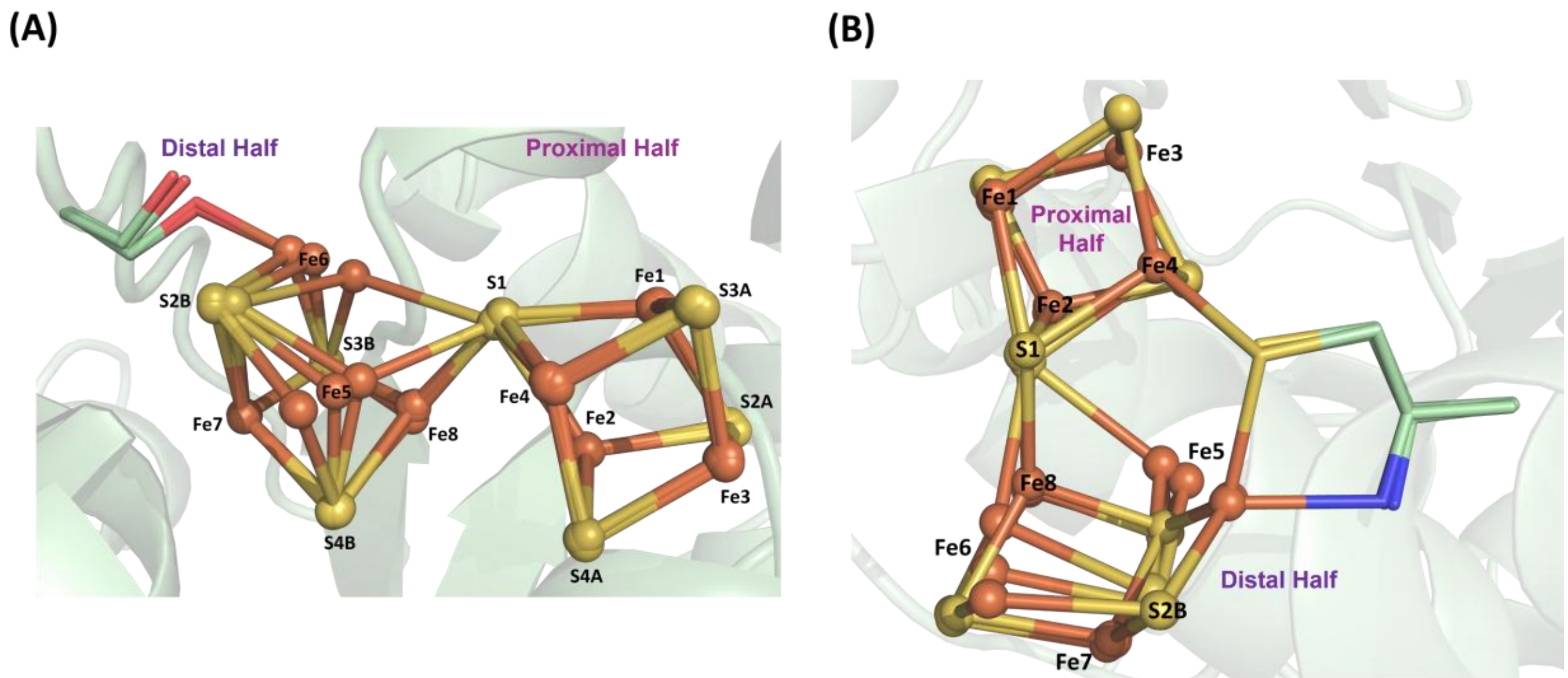


**Figure S1.** Overlay of the different redox states of P-cluster with the atomic labels of the cluster in 2 different orientations and proximity to FeMoco obtained in this work. (A) Front view of the cluster with **β-Ser188** shown (B) P-cluster rotated 90º clockwise to show top-down view of the cluster with **α-Cys88**.

### S3. Solvent Channels in MoFe Crystals

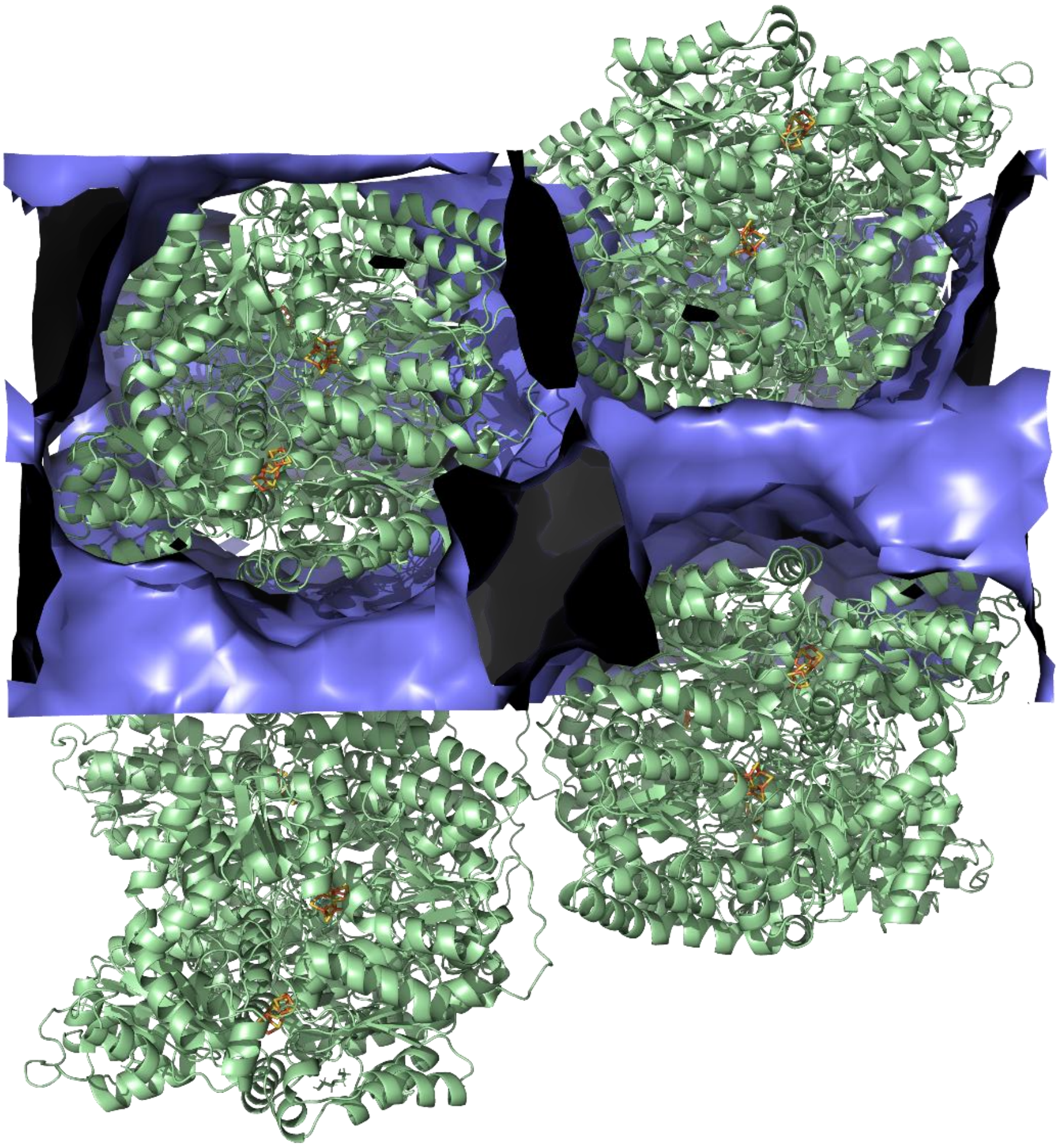

**Figure S2.** Solvent channels (purple) within the lattice of MoFe crystals (light green) calculated using MAP_CHANNELS.[16] The diameter of the narrowest point in the solvent channel network is 10 Å, similar to *E. coli* [NiFe]-hydrogenase-1 channels.[17]

## S4. X-ray data collection and refinement statistics

**Table S1.** Statistics for MoFe protein poised at each potential.

| **PDB code** | 29UL | 29UP | 29UM | 29UN | 29UO |
|---|---|---|---|---|---|
| Potential vs SHE | **+150 mV** | **+50 mV** | **-150 mV** | **-200 mV** | **-400 mV** |
| Species | $P^{3+}$ | $P^{2+}$ | $P^{2+}$ | $P^{1+}$ | $P^{N}$ |
| Space group | *C*2 | $P2_1$ | $P2_1$ | $P2_1$ | $P2_1$ |
| Unit cell dimensions (Å) | a=151.85 b=73.35 c=211.95 β=104.42° | a=84.01 b=156.85 c=202.28 β=90° | a=84.06 b=156.58 c=202.20 β=89.98° | a=84.14 b=156.82 c=202.32 β=89.99° | a=84.27 b=156.70 c=202.04 β=90.07° |
| Resolution (Å) | 52.71 – 2.50 (2.55 – 2.50) | 156.81-1.90 (1.93-1.90) | 156.58-1.90 (1.93-1.90) | 202.22-2.00 (2.03-2.00) | 156.70-1.90 (1.93-1.90) |
| Total reflections | 550236 (32133) | 2861493 (140640) | 2873477 (145536) | 2460563 (111991) | 2878053 (146493) |
| Unique reflections | 78429 (4454) | 402073 (19663) | 404327 (19754) | 348712 (17042) | 409971 (20212) |
| Completeness (%) | 99.8 (99.7) | 98.0 (96.8) | 98.7 (97.6) | 99.1 (98.3) | 99.8 (99.3) |
| Multiplicity | 7.0 (7.2) | 7.1 (7.2) | 7.1 (7.4) | 7.1 (6.6) | 7.0 (7.2) |
| $\langle I/\sigma \rangle$ | 6.8 (0.4) | 5.3 (0.6) | 5.7 (0.9) | 5.5 (0.8) | 8.0 (1.3) |
| $R_{merge}$ (%) | 0.227 (4.606) | 0.200 (3.030) | 0.153 (1.658) | 0.202 (1.997) | 0.160 (2.401) |
| $R_{pim}$ (%) | 0.092 (1.832) | 0.081 (1.215) | 0.062 (0.654) | 0.082 (0.842) | 0.064 (0.946) |
| $CC_{1/2}$ | 0.994 (0.449) | 0.994 (0.418) | 0.995 (0.776) | 0.990 (0.671) | 0.995 (0.586) |
| **Refinement** | | | | | |
| $R_{work}/R_{free}$ | 0.2375 / 0.2670 (0.4964 / 0.4870) | 0.1984 / 0.2272 (0.4440 / 0.4299) | 0.1829 / 0.2054 (0.3606 / 0.3622) | 0.1965 / 0.2227 (0.4115 / 0.3982) | 0.1697 / 0.1939 (0.3382 / 0.3555) |
| No. of atoms | 16073 | 33479 | 33897 | 33678 | 34147 |
| Macromolecule | 15950 | 31890 | 31884 | 31884 | 31884 |
| Solvent | 16 | 1248 | 1700 | 1466 | 1947 |
| **Average B-factors** | | | | | |
| Macromolecule | 84.97 | 40.29 | 38.88 | 41.46 | 35.65 |
| Solvent | 57.83 | 35.35 | 36.62 | 37.19 | 34.65 |
| RMSD bond lengths (Å) | 0.004 | 0.012 | 0.007 | 0.005 | 0.006 |
| RMSD angles (°) | 0.76 | 1.03 | 0.84 | 0.73 | 0.86 |
| Ramachandran plot favored / outliers (%) | 95.78 / 0.10 | 96.7 / 0.2 | 96.9 / 0.2 | 97.14 / 0.13 | 97.3 / 0.1 |
| Rotamer Outliers (%) | 0.46 | 0.41 | 0.32 | 0.38 | 0.35 |
| Clashscore | 8.92 | 2.93 | 2.42 | 1.93 | 2.28 |
| Molprobity Score | 1.77 | 1.29 | 1.20 | 1.11 | 1.14 |

Values in parenthesis refer to highest resolution shell.

## S5. Images of MoFe protein crystals and microcrystals

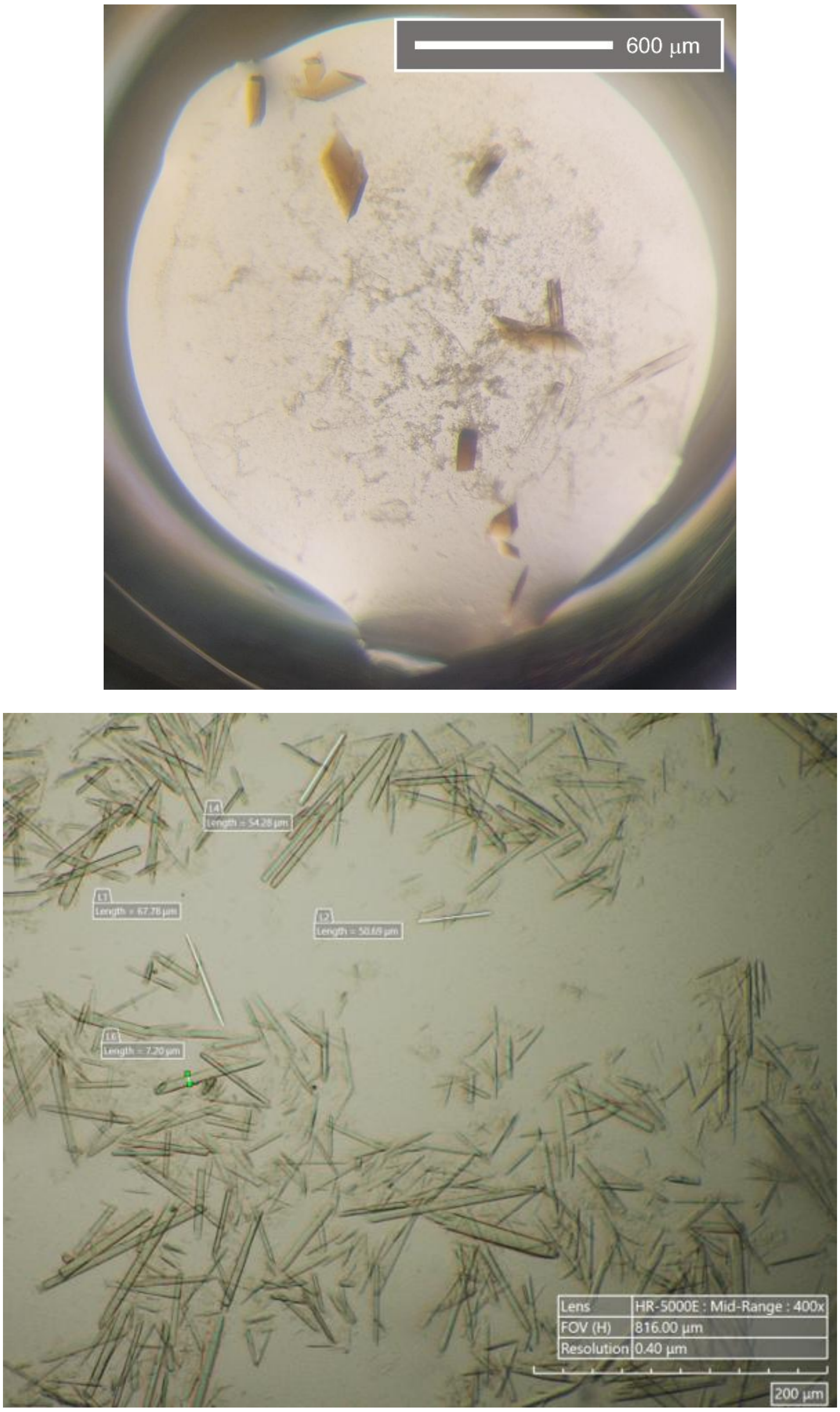


**Figure S3.** Crystals and microcrystals of MoFe used in this work. (top) crystals for cryogenic X-ray diffraction measurements grown using the sitting vapor diffusion method; (bottom) Microcrystals grown using modified vapor diffusion conditions described in methods. Scale bars indicate the size of the crystals.

## S6. Cyclic Voltammetry and $E_{1/2}$ of Mediators

**Table S2.** Mediators and their midpoint potentials in the MoFe cryo-buffer at pH 6. All potentials are reported vs SHE.

| Mediators | Phenazine methosulfate | Methylene blue | Flavin mononucleotide | Phenosafranin | Methyl viologen |
|---|---|---|---|---|---|
| $E_{1/2}$ | + 160 mV | +60 mV | -150 mV | -200 mV | -400 mV |

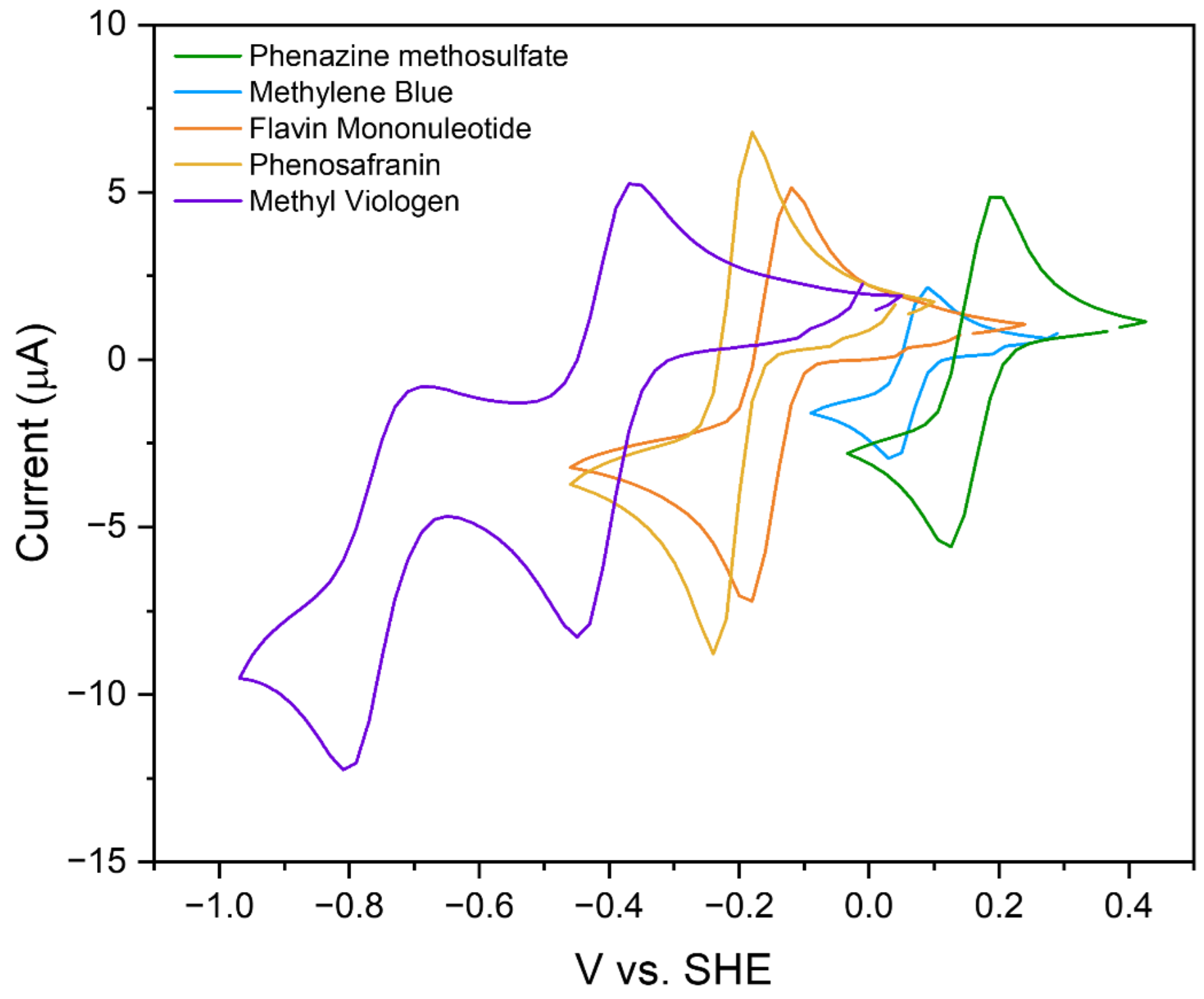


**Figure S4.** Overlay of cyclic voltammograms of 1 mM phenazine methosulfate, methylene blue, flavin mononucleotide, phenosafranin and methyl viologen recorded at 20 mV/s in the cryo-buffer with 0.1 M NaCl as electrolyte.

## S7. Additional EPR Spectral data

### S7.1 Buffer Control Spectra

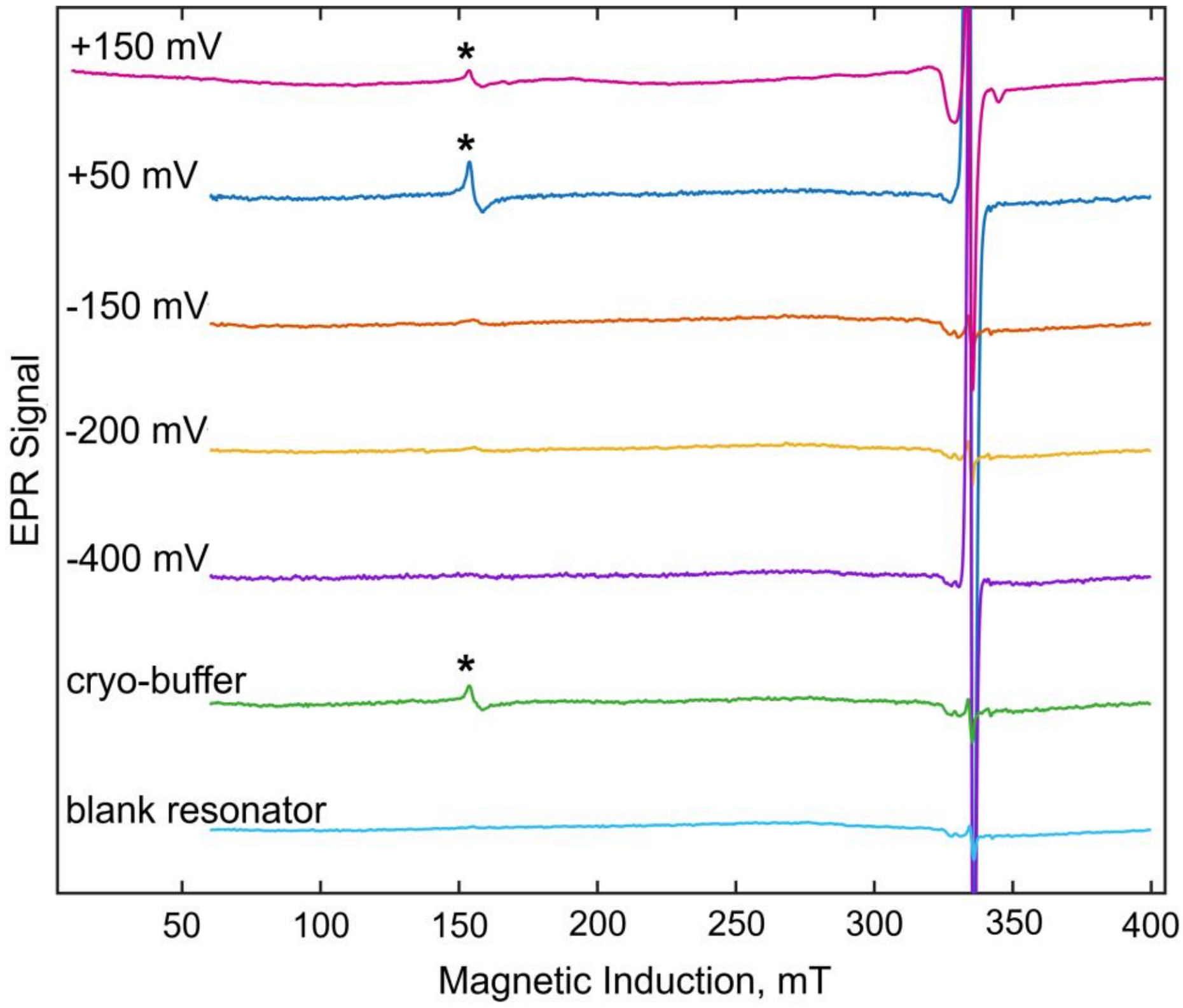


**Figure S5.** Control X-band perpendicular mode CW-EPR spectra used in subtraction for Fig 2A (main text). The small feature at $g = 4.3$ (*) is from trace amounts of aqueous $Fe^{3+}$ and the sharp intense $g \sim 2.00$ feature arises from radicals of methylene blue (MB)[18] and methyl viologen (MV).[19] In this field range, contributions from resonator and cryostat backgrounds are less intense. Microwave power of 10 mW was used for +150 mV buffer, while the rest was measured using 80 uW.

**S7.2 Resting State MoFe Protein Microcrystal Spectrum**

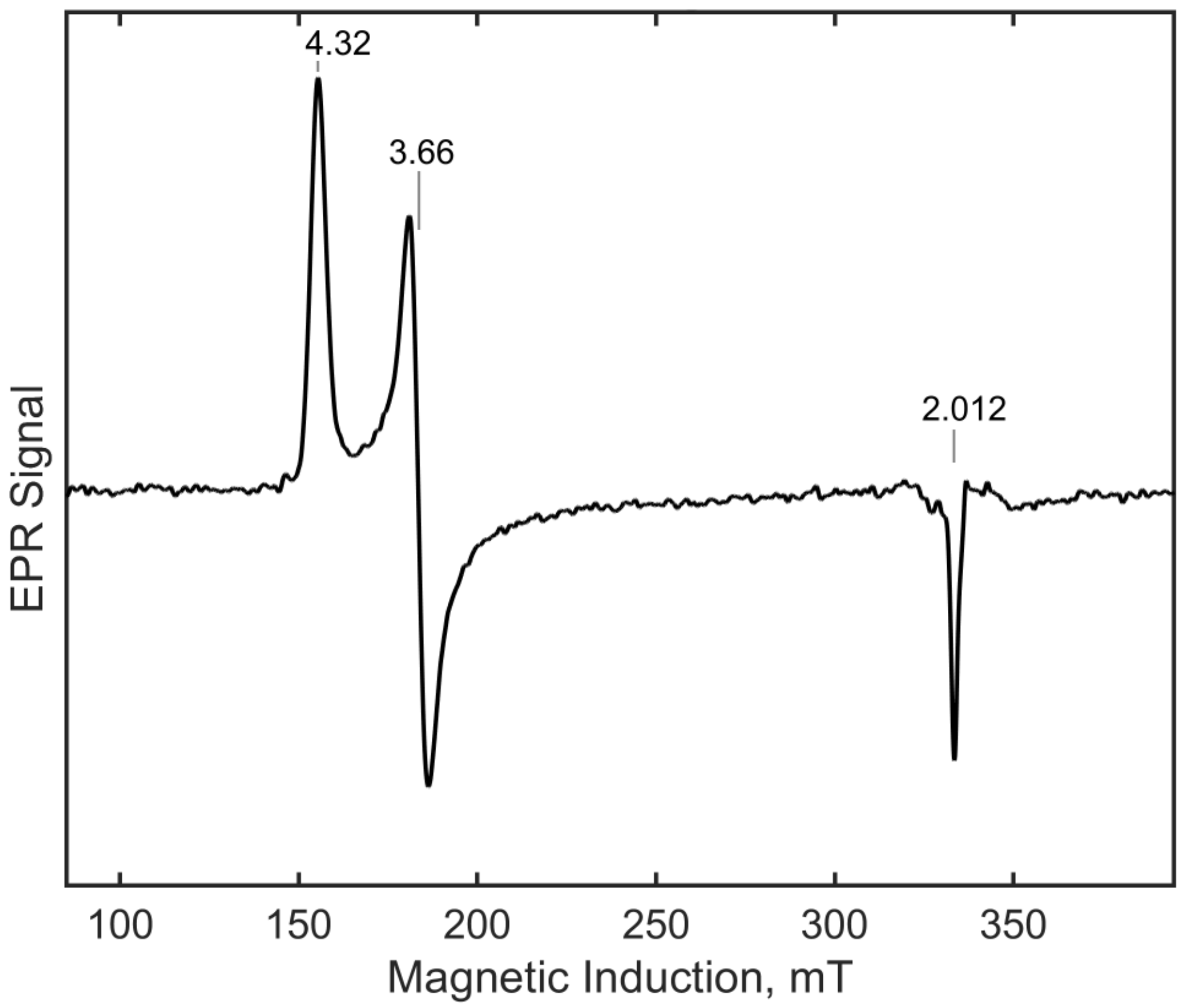


**Figure S6.** Measurement of perpendicular mode EPR of resting state MoFe protein microcrystals (obtained by soaking in cryo-buffer with 5mM dithionite) at 5 K exhibiting the rhombic, $S$ = 3/2 signal with $g$ = 4.32, 3.66, 2.01 similar to literature.[20] (microwave frequency = 9.3878 GHz, microwave power = 50 uW, field modulation = 0.6 mT, with 3 sweeps of 60 seconds, averaged, at 310 mT).

## S7.3 Estimation of Population using Electron Density Maps and EPR simulations of relative signal intensity of $P^{1+}$

Table S3 shows the relative populations of P-cluster states calculated from the X-ray structures.

**Table S3.** Population of P-cluster redox states present at the different electrochemical poised levels as determined from X-ray structures. $P^{1+}$ populations were estimated using difference in peak heights in the mFo-DFc density map. All other states showed no evidence of mixed populations after multiple rounds of structural refinement.

| Potential (mV) | Population of Species (%) | | | |
|---|---|---|---|---|
| | **$P^N$** | **$P^{1+}$** | **$P^{2+}$** | **$P^{3+}$** |
| **+150**** | 0 | 0 | 0 | 100 |
| **+50** | 0 | 0 | 100 | 0 |
| **-150** | 0 | 0 | 100 | 0 |
| **-200** | 10 | 90 | 0 | 0 |
| **-400** | 100 | 0 | 0 | 0 |

**Owing to the much lower resolution of this dataset, possible residual mixed species may not be resolved in the current maps, and the apparent single-species assignment should therefore be interpreted with appropriate caution.

There are several limitations to absolute EPR spin quantification for a protein microcrystal slurry. First, the amount of microcrystals, i.e. total protein concentration, per sample tube is not clearly defined. Second, due to variations in density of packing of microcrystals from one tube to another and within each tube, the suspension of the microcrystals is not exactly reproducible over the inhomogeneous $B_1$ field distribution of the resonator. At potentials in the range -150 mV to -400 mV, we assume that the FeMoco center is entirely in the $M^N$, $S$=3/2 state. Thus, the signal from $M^N$ is used as an internal reference to enable calculation of the relative change in $P^{1+}$ signal intensity between -150 and -200 mV microcrystal preparations.

A 5-fold relative decrease in the amount of the $P^{1+}$ state at -150 mV relative to -200 mV is calculated from perpendicular-mode EPR simulations, using the ‘pepper’ function in EasySpin.[21] Signals analyzed were the $S$ = 1/2 $P^{1+}$ state and $S$ = 3/2 FeMoco species ($M^N$). $M^N$ is simulated as $S$= 3/2, with D = 10 $cm^{-1}$, E/D = 0.057 and $g$ = 2.01, which generates the observed $S_{eff}$=1/2, $g_{obs}$= [4.32 3.66 2.00]. The $P^{1+}$ state is simulated as $S$= 1/2, and $g$ = [2.05 1.95 1.82]. For analysis, the $M^N$ signal is quantified by the intensity of the central $g$-value, $g_{obs}$ = 3.66, windowed over a narrow portion of the spectrum to limit baseline artefact contributions. The $P^{1+}$ state is quantified by windowing over $g_{obs}$ = 1.82, since this feature is spectrally-isolated. Despite spectroscopic evidence for a low contribution of $P^{1+}$ at -150 mV, there was no clear evidence of this state in the electron density maps (Table S3).

## S7.4 Parallel Mode EPR Spectra

Additional measurements in parallel mode show that of the $P^{2+}$ signal decreases during progressive reduction from +50 mV to -400 mV. Due to the signal-to-noise and different resonator used compared to perpendicular mode, quantitative values were not determined. For reference, sample-to-sample variation of the resting state *S*=3/2 signal was +/-10% between the -150 mV, -200 mV and -400 mV samples.

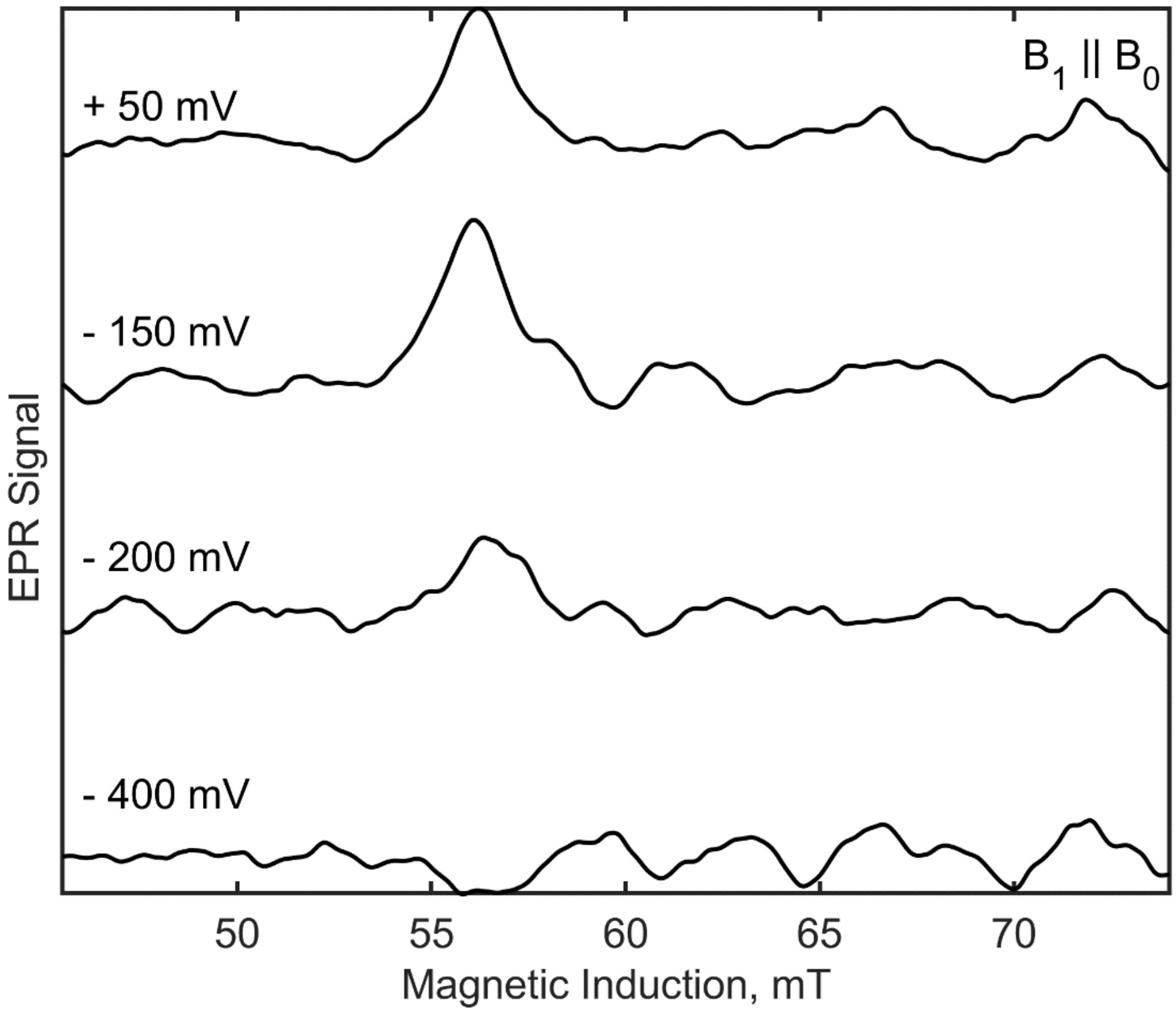


**Figure S7.** Measurement of parallel mode EPR in the region of the $P^{2+}$ signals, *g* = 11.9, over the different potentials at 10 K (microwave power = 100 mW, modulation amplitude = 0.5 mT, with 20-60 sweeps of 60 seconds, averaged, followed by a 9th-order baseline removal, and microwave frequencies of 9.3815, 9.3732, 9.3840, and 9.3899 GHz for +50 mV, -150 mV, -200 mV, and -400 mV, respectively).

## S7.5 Perpendicular Mode, Low-Field EPR Spectra

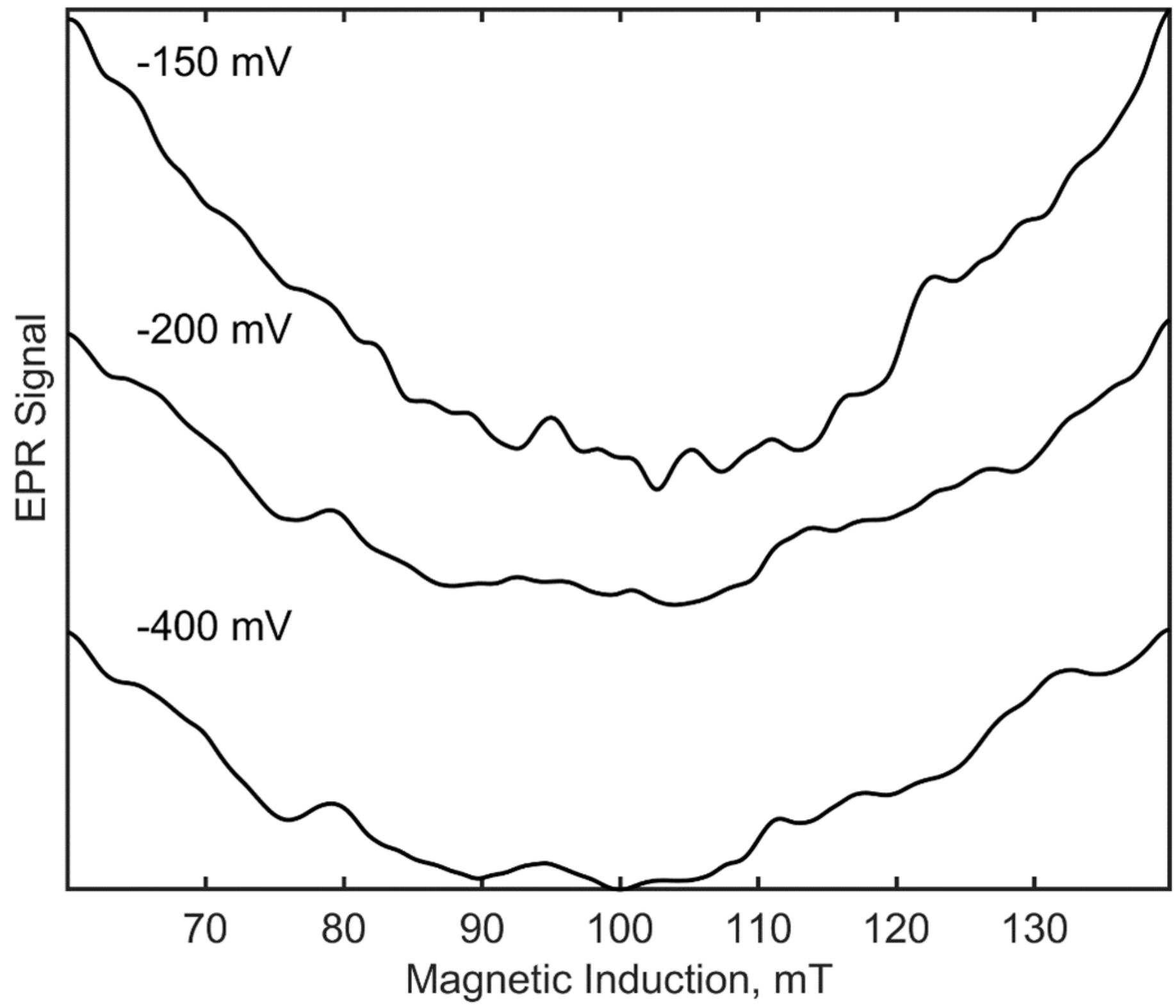


**Figure S8.** Measurement of perpendicular mode X-band CW-EPR in the region of the high spin *S*=7/2, *g* = 6.54 of $P^{1+}_{6.54}$ conformer (not observed) at 12 K (microwave power = 80 mW, modulation amplitude = 2 mT, with 4 sweeps of 72 seconds averaged and microwave frequencies = 9.3794, 9.393, & 9.3938 GHz for -150 mV, -200 mV, and -400 mV, respectively).

Similarly, the other rhombic *S*=1/2 conformer, $P^{1+}_{1.89}$ was also not observed in these potential range.

## S8. Additional Structural Data

### S8.1 2mFo-DFc Maps Around the P-Cluster

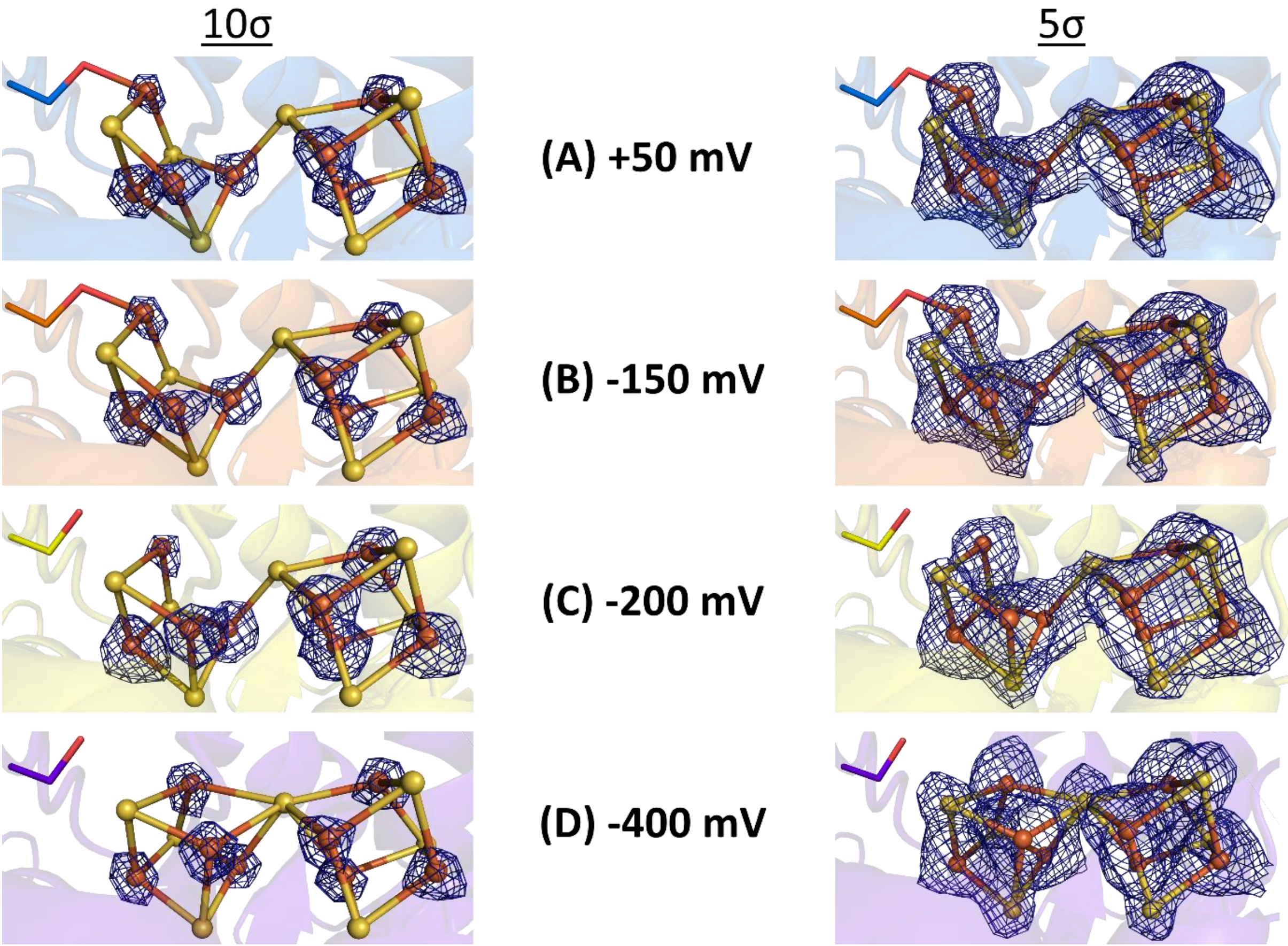


**Figure S9.** Maps of poised structures 2mFo-DFc maps (blue mesh) around the P-cluster contoured to 10σ and 5σ in PyMOL for the different poised structures of MoFe. Iron atoms are indicated in dark orange, sulfur in dark yellow, oxygen in red, and the carbon atoms colored respective to the different poises (blue = +50 mV, light orange = -150 mV, light yellow = -200 mV and purple = -400 mV).

## S8.2 2mFo-DFc Maps Around the M-Cluster

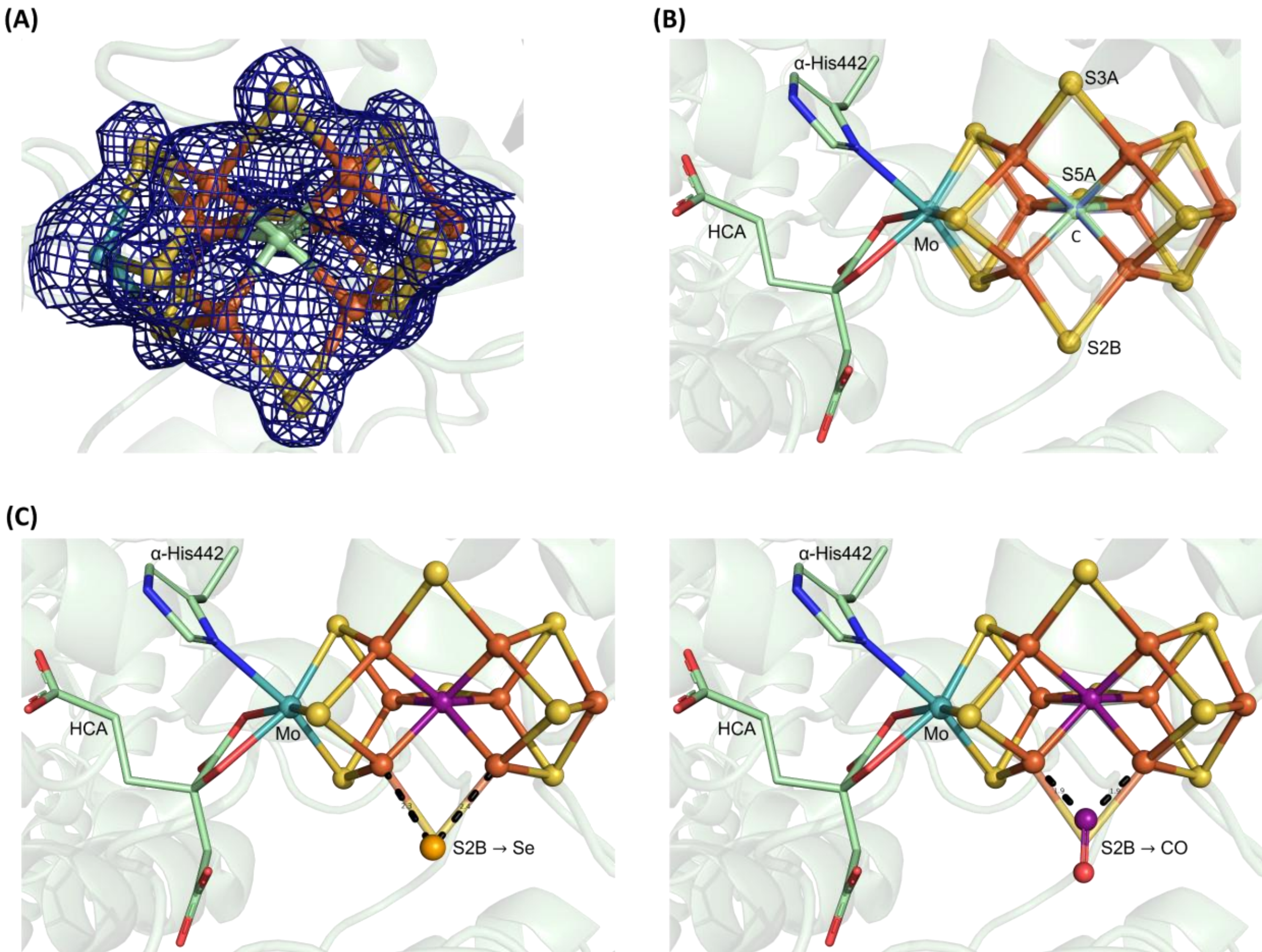


**Figure S10.** (A) Superimposed structures of the M-cluster at +50 mV, -150 mV, -200 mV and -400 mV mapped with electron density (2mFo-DFc) map of +50 mV at 5σ demonstrates the rigidity of the cluster for this potential range. (B) Overlay of 12 structures of *A. vinelandii* MoFe protein collected under oxidized and reduced or turnover conditions reported in literature - PDB Codes: 3U7Q, 1M1N, 4TKU, 6BBL, 6VXT, 8DBX, 9CJD, 4XPI, 2MIN, 6CDK, 3K1A and 8DBY. (C) Structures recorded under turnover when treated with KSeCN and CO (PDB: 4TKV and 5BVG). Iron atoms are indicated in dark orange, sulfur in dark yellow, molybdenum in teal and carbon in green and purple.

No structural change is observed at the M-cluster at any of the potentials applied (Fig S10A). This is consistent with the M-cluster in 12 available structures of *A. vinelandii.* MoFe protein collected under oxidized and reduced or turnover conditions (Fig S10B). Notable exceptions are MoFe protein crystallized from a turnover mixture in the presence of KSeCN and CO (Fig S10C).

## S8.3 Comparison of P-Cluster and local environment between redox states

**Table S4**. Averaged Values of Bond Distance between **β-Ser[188]** and **Fe6**, and **α-Cys(N)[88]** and **Fe5** for all chains, where standard deviation was calculated using the population formula (STDEV.P)

| Potential (mV) | +150 mV | +50 mV | -150 mV | -200 mV | -400 mV |
|---|---|---|---|---|---|
| β-$Ser^{188}$ – Fe6 (Å) | 2.02 ± 0.1 | 1.95 ± 0.04 | 1.96 ± 0.03 | 2.59 ± 0.02 | 3.41± 0.09 |
| S1 – Fe6 (Å) | 3.69 ± 0.01 | 3.81 ± 0.04 | 3.82 ± 0.01 | 3.34 ± 0.01 | 2.56 ± 0.04 |
| α-$Cys(N)^{88}$ – Fe5 (Å) | 2.42 ± 0.01 | 2.25 ± 0.02 | 2.29 ± 0.04 | 2.84 ± 0.04 | 3.39 ± 0.02 |
| S1 – Fe5 (Å) | 3.62 ± 0.1 | 3.74 ± 0.09 | 3.68 ± 0.06 | 3.01 ± 0.01 | 2.48 ± 0.02 |

Chain E

**Table S5**. Selected bond lengths and angles of the P-Cluster and first coordination sphere for Chain E.

| Bond Length (Å) | +50 mV | -150 mV | -200 mV | -400 mV |
|---|---|---|---|---|
| β-$Ser^{188}$ – Fe6 | 1.90 | 1.92 | 2.61 | 3.50 |
| S1 – Fe6 | 3.78 | 3.81 | 3.33 | 2.52 |
| α-$Cys^{153}$ – Fe6 | 2.40 | 2.35 | 2.33 | 2.34 |
| S1 – Fe5 | 3.65 | 3.70 | 3.00 | 2.44 |
| α-$Cys^{88}$ – Fe5 | 2.24 | 2.33 | 2.19 | 2.26 |
| α-$Cys(N)^{88}$ – Fe5 | 2.26 | 2.24 | 2.89 | 3.40 |
| β-$Cys^{95}$ – Fe8 | 2.29 | 2.32 | 2.33 | 2.38 |
| β-$Cys^{70}$ – Fe7 | 2.42 | 2.37 | 2.36 | 2.30 |
| α-$Cys^{88}$ – Fe4 | 2.36 | 2.30 | 2.31 | 2.38 |
| α-$Cys^{62}$ – Fe3 | 2.32 | 2.31 | 2.29 | 2.27 |
| β-$Cys^{95}$ – Fe2 | 2.23 | 2.26 | 2.30 | 2.31 |
| β-$C(N)^{95}$ – Fe2 | 3.30 | 3.26 | 3.03 | 3.24 |
| α-$Cys^{154}$ – Fe1 | 2.37 | 2.39 | 2.40 | 2.36 |

| Bond Angle (°) | +50 mV | -150 mV | -200 mV | -400 mV |
|---|---|---|---|---|
| Fe6 – S2B – Fe5 | 112.64 | 114.38 | 90.05 | 68.21 |
| Fe6 – S1 – Fe5 | 61.91 | 62.02 | 61.23 | 62.78 |
| S2B – Fe6 – S1 | 87.74 | 87.76 | 96.29 | 108.77 |
| S2B – Fe5 – S1 | 91.43 | 91.35 | 107.47 | 115.61 |
| Fe5 – α-$Cys^{88}$ – Fe4 | 106.18 | 104.54 | 89.17 | 77.44 |
| α-$C(N)^{88}$ – Fe5 – α-$Cys^{88}$ | 79.69 | 80.72 | 67.79 | 58.71 |

Chain A

**Table S6**. Selected bond lengths and angles of the P-Cluster and first coordination sphere for Chain A.

| Bond Length (Å) | +50 mV | -150 mV | -200 mV | -400 mV |
|---|---|---|---|---|
| β-Ser[188] – Fe6 | 1.99 | 1.97 | 2.57 | 3.27 |
| S1 – Fe6 | 3.84 | 3.82 | 3.35 | 2.62 |
| α-Cys[153] – Fe6 | 2.36 | 2.39 | 2.36 | 2.32 |
| S1 – Fe5 | 3.90 | 3.62 | 3.03 | 2.49 |
| α-Cys[88] – Fe5 | 2.28 | 2.30 | 2.15 | 2.29 |
| α-Cys(N)[88] – Fe5 | 2.28 | 2.34 | 2.79 | 3.37 |
| β-Cys[95] – Fe8 | 2.35 | 2.25 | 2.37 | 2.29 |
| β-Cys[70] – Fe7 | 2.39 | 2.38 | 2.34 | 2.37 |
| α-Cys[88] – Fe4 | 2.24 | 2.27 | 2.31 | 2.35 |
| α-Cys[62] – Fe3 | 2.27 | 2.28 | 2.30 | 2.26 |
| β-Cys[95] – Fe2 | 2.32 | 2.30 | 2.29 | 2.32 |
| β-C(N)[95] – Fe2 | 3.24 | 3.23 | 3.20 | 3.22 |
| α-Cys[154] – Fe1 | 2.20 | 2.27 | 2.35 | 2.27 |

| Bond Angle (º) | +50 mV | -150 mV | -200 mV | -400 mV |
|---|---|---|---|---|
| Fe6 – S2B – Fe5 | 114.43 | 112.47 | 94.45 | 71.46 |
| Fe6 – S1 – Fe5 | 56.04 | 62.06 | 63.56 | 63.37 |
| S2B – Fe6 – S1 | 88.01 | 87.97 | 93.62 | 107.20 |
| S2B – Fe5 – S1 | 91.53 | 94.13 | 103.95 | 114.11 |
| Fe5 – α-Cys[88] – Fe4 | 105.88 | 101.00 | 92.29 | 76.27 |
| α-C(N)[88] – Fe5 – α-Cys[88] | 82.23 | 81.67 | 69.50 | 61.22 |

Chain C

**Table S7**. Selected bond lengths and angles of the P-Cluster and first coordination sphere for Chain C.

| Bond Length (Å) | +50mV | -150mV | -200mV | -400mV |
|---|---|---|---|---|
| β-Ser$^{188}$ – Fe6 | 1.91 | 2.00 | 2.60 | 3.48 |
| S1 – Fe6 | 3.77 | 3.82 | 3.33 | 2.55 |
| α-Cys$^{153}$ – Fe6 | 2.38 | 2.38 | 2.36 | 2.33 |
| S1 – Fe5 | 3.70 | 3.63 | 3.00 | 2.49 |
| α-Cys$^{88}$ – Fe5 | 2.28 | 2.22 | 2.22 | 2.29 |
| α-Cys(N)$^{88}$ – Fe5 | 2.23 | 2.32 | 2.87 | 3.42 |
| β-Cys$^{95}$ – Fe8 | 2.34 | 2.32 | 2.35 | 2.32 |
| β-Cys$^{70}$ – Fe7 | 2.45 | 2.39 | 2.29 | 2.39 |
| α-Cys$^{88}$ – Fe4 | 2.34 | 2.36 | 2.28 | 2.36 |
| α-Cys$^{62}$ – Fe3 | 2.28 | 2.28 | 2.32 | 2.29 |
| β-Cys$^{95}$ – Fe2 | 2.23 | 2.26 | 2.25 | 2.33 |
| β-C(N)$^{95}$ – Fe2 | 3.22 | 3.25 | 3.20 | 3.22 |
| α-Cys$^{154}$ – Fe1 | 2.36 | 2.32 | 2.43 | 2.35 |

| Bond Angle (°) | +50mV | -150mV | -200mV | -400mV |
|---|---|---|---|---|
| Fe6 – S2B – Fe5 | 112.23 | 113.27 | 89.58 | 68.50 |
| Fe6 – S1 – Fe5 | 61.43 | 61.81 | 61.20 | 61.72 |
| S2B – Fe6 – S1 | 90.22 | 87.23 | 97.14 | 109.02 |
| S2B – Fe5 – S1 | 92.00 | 92.45 | 108.12 | 114.66 |
| Fe5 – α-Cys$^{88}$ – Fe4 | 105.26 | 102.99 | 89.73 | 76.34 |
| α-C(N)$^{88}$ – Fe5 – α-Cys$^{88}$ | 79.94 | 79.07 | 69.59 | 57.09 |

Chain G

**Table S8**. Selected bond lengths and angles of the P-Cluster and first coordination sphere for Chain G.

| Bond Length (Å) | +50mV | -150mV | -200mV | -400mV |
|---|---|---|---|---|
| β-Ser$^{188}$ – Fe6 | 1.99 | 1.96 | 2.58 | 3.40 |
| S1 – Fe6 | 3.85 | 3.83 | 3.33 | 2.56 |
| α-Cys$^{153}$ – Fe6 | 2.40 | 2.42 | 2.36 | 2.37 |
| S1 – Fe5 | 3.72 | 3.77 | 3.00 | 2.48 |
| α-Cys$^{88}$ – Fe5 | 2.99 | 2.29 | 2.19 | 2.30 |
| α-Cys(N)$^{88}$ – Fe5 | 2.24 | 2.25 | 2.81 | 3.37 |
| β-Cys$^{95}$ – Fe8 | 2.34 | 2.29 | 2.36 | 2.26 |
| β-Cys$^{70}$ – Fe7 | 2.39 | 2.45 | 2.39 | 2.39 |
| α-Cys$^{88}$ – Fe4 | 2.24 | 2.23 | 2.33 | 2.30 |
| α-Cys$^{62}$ – Fe3 | 2.30 | 2.26 | 2.36 | 2.28 |
| β-Cys$^{95}$ – Fe2 | 2.31 | 2.26 | 2.29 | 2.28 |
| β-C(N)$^{95}$ – Fe2 | 3.20 | 3.22 | 3.23 | 3.20 |
| α-Cys$^{154}$ – Fe1 | 2.31 | 2.22 | 2.37 | 2.30 |

| Bond Angle (º) | +50mV | -150mV | -200mV | -400mV |
|---|---|---|---|---|
| Fe6 – S2B – Fe5 | 112.23 | 116.12 | 93.17 | 70.30 |
| Fe6 – S1 – Fe5 | 60.57 | 61.50 | 63.48 | 63.20 |
| S2B – Fe6 – S1 | 89.26 | 87.83 | 94.06 | 109.13 |
| S2B – Fe5 – S1 | 93.12 | 90.15 | 104.83 | 112.79 |
| Fe5 – α-Cys$^{88}$ – Fe4 | 103.96 | 106.17 | 90.81 | 76.99 |
| α-C(N)$^{88}$ – Fe5 – α-Cys$^{88}$ | 81.23 | 84.12 | 69.88 | 60.71 |

## S9. Comparison between published P-Cluster Redox States of MoFe protein and this work

### S9.2 $P^N$

**Table S9**. Selected bond lengths and angles of reported $P^N$ states in MoFe

| PDB | 29UO | 1M1N | 3K1A* | 3MIN | 6VXT | 3U7Q |
|---|---|---|---|---|---|---|
| **Resolution (Å)** | 1.90 | 1.16 | 2.23 | 2.03 | 1.74 | 1.00 |
| **Chain** | G | H | D | D | C | D |
| **Bond Lengths (Å)** | | | | | | |
| β-Ser[188] – Fe6 | 3.40 | 3.51 | 3.34 | 2.84 | 3.42 | 3.23 |
| S1 – Fe6 | 2.56 | 2.45 | 2.19 | 2.60 | 2.51 | 2.56 |
| α-Cys[153] – Fe6 | 2.37 | 2.31 | 2.70 | 2.36 | 2.43 | 2.26 |
| S1 – Fe5 | 2.48 | 2.43 | 2.16 | 2.31 | 2.42 | 2.63 |
| α-Cys[88] – Fe5 | 2.30 | 2.38 | 2.31 | 2.31 | 2.32 | 2.27 |
| α-Cys(N)[88] – Fe5 | 3.37 | 3.50 | 3.35 | 3.40 | 3.45 | 3.21 |
| **Bond Angle (º)** | | | | | | |
| Fe6 – S2B – Fe5 | 70.30 | 66.45 | 61.90 | 67.17 | 66.39 | 61.03 |
| Fe6 – S1 – Fe5 | 63.20 | 62.89 | 63.12 | 61.53 | 61.75 | 58.51 |
| S2B – Fe6 – S1 | 109.13 | 111.01 | 111.03 | 109.18 | 110.33 | 116.95 |
| S2B – Fe5 – S1 | 112.79 | 113.87 | 111.55 | 116.77 | 113.94 | 118.10 |
| Fe5 – α-Cys[88] – Fe4 | 76.99 | 75.51 | 62.56 | 81.56 | 76.22 | 79.83 |
| α-C(N)[88] – Fe5 – α-Cys[88] | 60.71 | 59.52 | 60.98 | 60.18 | 56.54 | 62.78 |

* - Proximal side of the cluster to FeMoco is distorted

## S9.2 $P^{1+}$

**Table S10**. Selected bond lengths and angles of reported $P^{1+}$ state in MoFe

| PDB | 29UN | 29UP | 6CDK* | |
|---|---|---|---|---|
| **Redox State** | $P^{1+}$ | $P^{2+}$ | $P^{1+}$ | |
| **Resolution (Å)** | 2.00 | 1.90 | 2.10 | |
| **Chain** | G | G | A | A |
| **Bond Lengths (Å)** | | | | |
| β-Ser$^{188}$ – Fe6 | 2.58 | 1.99 | 2.02 | 2.59 |
| S1 – Fe6 | 3.33 | 3.85 | 3.92 | 2.98 |
| α-Cys$^{153}$ – Fe6 | 2.36 | 2.40 | 2.40 | 2.28 |
| S1 – Fe5 | 3.00 | 3.72 | 3.45 | 2.74 |
| α-Cys$^{88}$ – Fe5 | 2.19 | 2.99 | 2.35 | 2.31 |
| α-Cys(N)$^{88}$ – Fe5 | 2.81 | 2.24 | 2.25 | 3.08 |
| **Bond Angle (º)** | 29UN | 29UP | 6CDK | |
| Fe6 – S2B – Fe5 | 93.17 | 112.23 | 114.45 | 77.04 |
| Fe6 – S1 – Fe5 | 63.48 | 60.57 | 60.81 | 62.54 |
| S2B – Fe6 – S1 | 94.06 | 89.26 | 83.02 | 103.13 |
| S2B – Fe5 – S1 | 104.83 | 93.12 | 97.68 | 112.60 |
| Fe5 – α-Cys$^{88}$ – Fe4 | 90.81 | 103.96 | 111.31 | 89.53 |
| α-C(N)$^{88}$ – Fe5 – α-Cys$^{88}$ | 69.88 | 81.23 | 84.36 | 65.91 |

*Reported structure was subsequently determined by QM/MM calculations[22] to be a mixture of $P^{1+}$ and $P^{2+}$.

## S9.3 Is the P-cluster of *M. infernus* MoFe protein also in the $P^{1+}$ state?

A recent preprint presents the X-ray crystal structure of MoFe protein of nitrogenase from thermophilic methanogen *M. infernus* which the authors assign as the $P^{1+}$ state.[23] Identification of the $P^{1+}$ state was based on the observation that the structure of the P-cluster differed from the well-characterized $P^{N}$ and $P^{2+}$ states. Since there was no independent spectroscopic verification of the oxidation state of the P-cluster and the precise redox conditions applied to the crystals were unclear, the assignment of the observed structure as $P^{1+}$ should be treated with caution. Coordinates of this enzyme are not available for comparison at the time of writing.

## S9.4 $P^{2+}$

**Table S11**. Selected bond lengths and angles of reported $P^{2+}$ states in MoFe

| **PDB** | 29UP | 2MIN | 3U7Q | 4ND8 | 4WN9 | 4XPI |
|---|---|---|---|---|---|---|
| **Resolution (Å)** | 1.90 | 2.03 | 1.00 | 2.00 | 1.90 | 1.97 |
| **Chain** | G | A | B | C | C | C |
| **Bond Lengths (Å)** | | | | | | |
| β-Ser$^{188}$ – Fe6 | 1.99 | 1.90 | 1.91 | 2.23 | 2.05 | 1.90 |
| S1 – Fe6 | 3.85 | 3.96 | 3.86 | 3.97 | 3.46 | 4.15 |
| α-Cys$^{153}$ – Fe6 | 2.40 | 2.27 | 2.39 | 2.50 | 2.34 | 2.33 |
| S1 – Fe5 | 3.72 | 3.88 | 3.80 | 3.77 | 3.54 | 3.87 |
| α-Cys$^{88}$ – Fe5 | 2.99 | 2.43 | 2.31 | 2.55 | 2.31 | 2.46 |
| α-Cys(N)$^{88}$ – Fe5 | 2.24 | 2.15 | 2.12 | 2.29 | 2.23 | 2.39 |

| **Bond Angle (º)** | | | | | | |
|---|---|---|---|---|---|---|
| Fe6 – S2B – Fe5 | 112.23 | 113.97 | 119.25 | 117.97 | 95.52 | 122.08 |
| Fe6 – S1 – Fe5 | 60.57 | 58.60 | 62.47 | 57.03 | 61.71 | 56.69 |
| S2B – Fe6 – S1 | 89.26 | 89.86 | 85.85 | 87.02 | 100.20 | 86.33 |
| S2B – Fe5 – S1 | 93.12 | 93.80 | 87.89 | 93.47 | 99.47 | 94.89 |
| Fe5 – α-Cys$^{88}$ – Fe4 | 103.96 | 111.16 | 105.96 | 102.00 | 93.68 | 98.30 |
| α-C(N)$^{88}$ – Fe5 – α-Cys$^{88}$ | 81.23 | 84.50 | 84.02 | 74.66 | 82.33 | 77.12 |

## S10. Calculated Iron Anomalous Maps

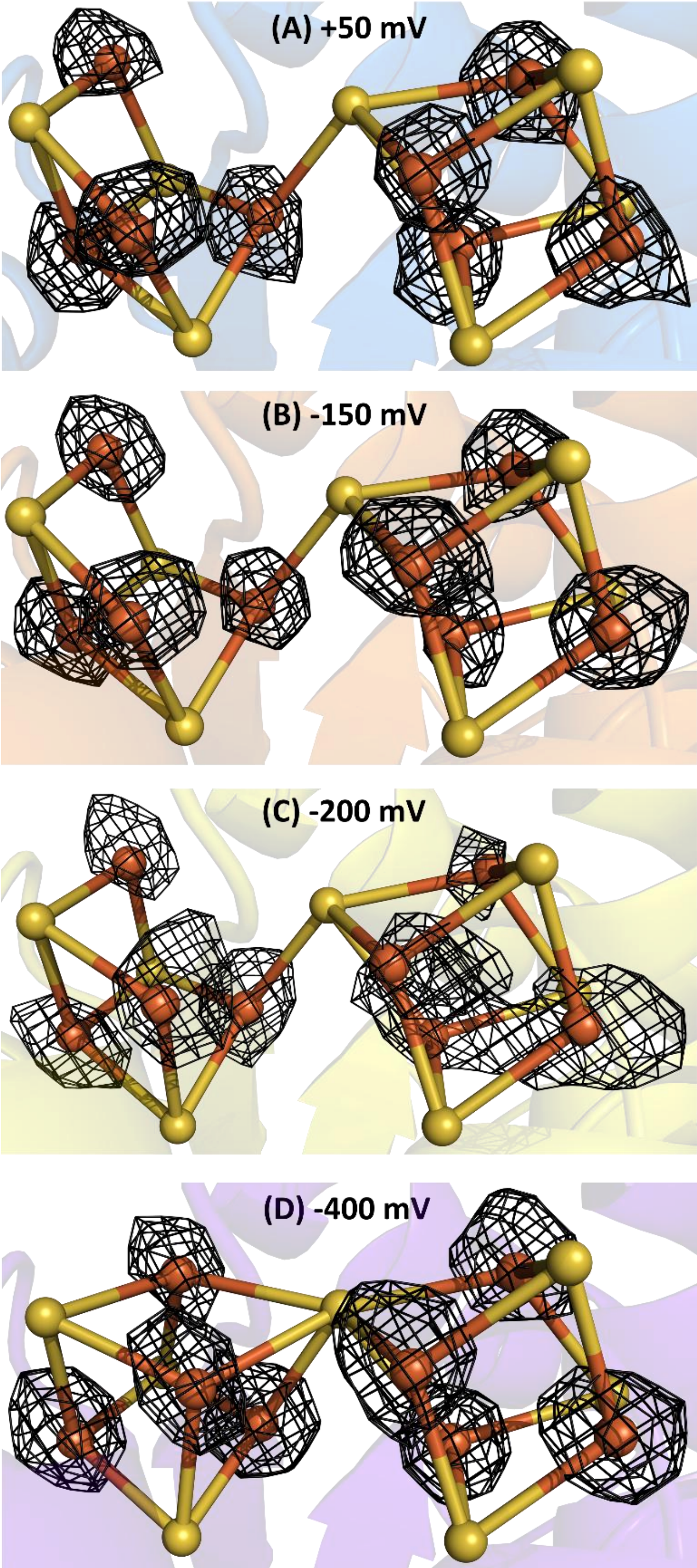


**Figure S11.** Iron anomalous difference maps (black mesh) calculated using data collected at 0.95 Å highlight the iron atom (dark orange) positions in the P-cluster of MoFe protein poised at each different potential (A) +50 mV, (B) -150 mV and (D) -400 mV contoured at 5σ, and (C) -200 mV contoured at 3.8σ.

### S11. Calculated Difference Maps

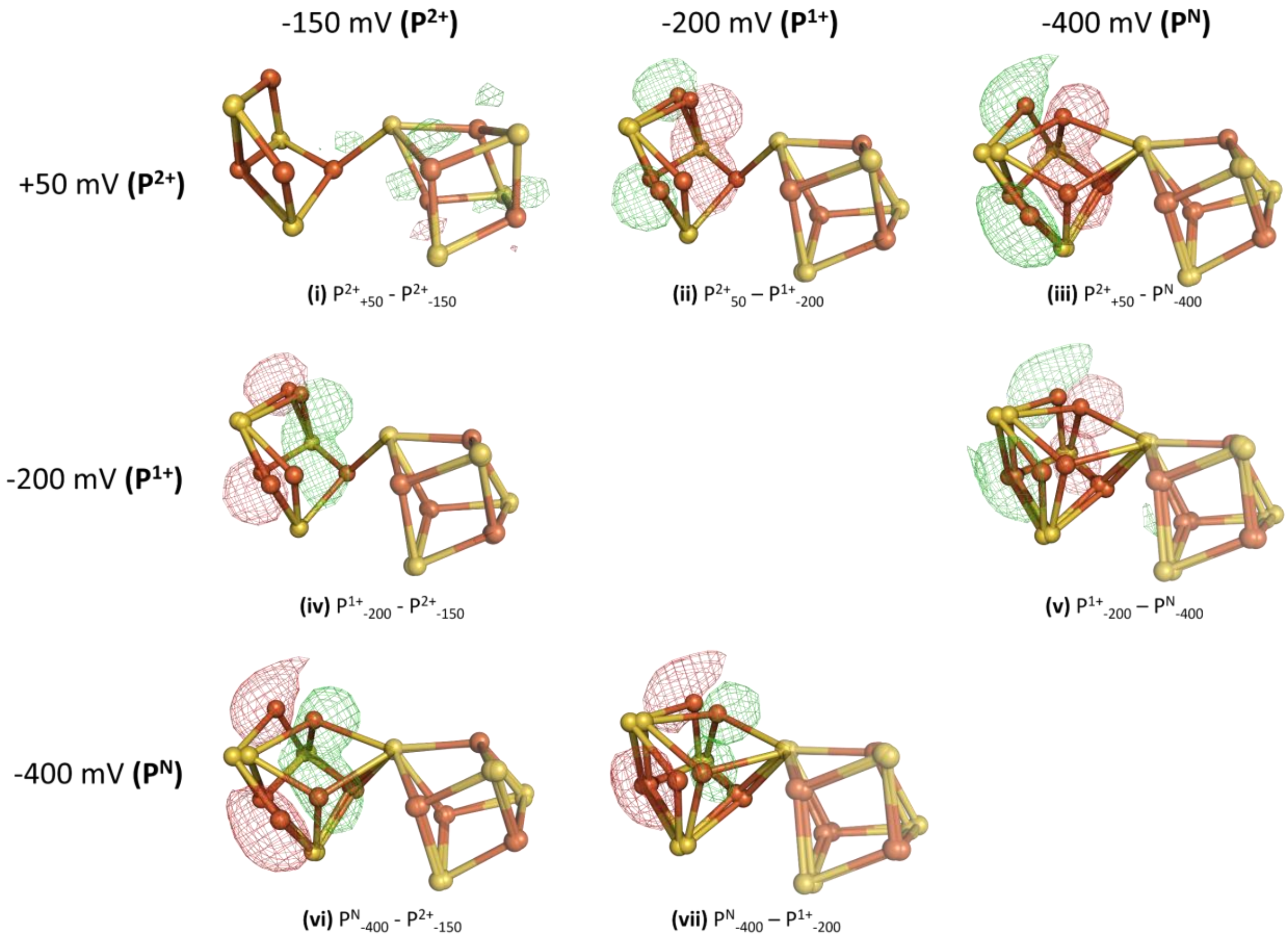


**Figure S12.** Difference electron density maps ($mF_o$-$DF_o$) calculated to compare how the electron density distribution around the P-cluster varies with each redox poise. The rigid half of the cluster has been faded for clarity. Atom labels are: iron (orange) and sulfur (yellow). Each column and row indicate the corresponding two models used to calculate each map, with green mesh signifying positive difference density and red mesh corresponding to negative difference density. All maps are contoured at $\geq 8\sigma$.

## S12. Root Mean Square Deviation of Poised Structures

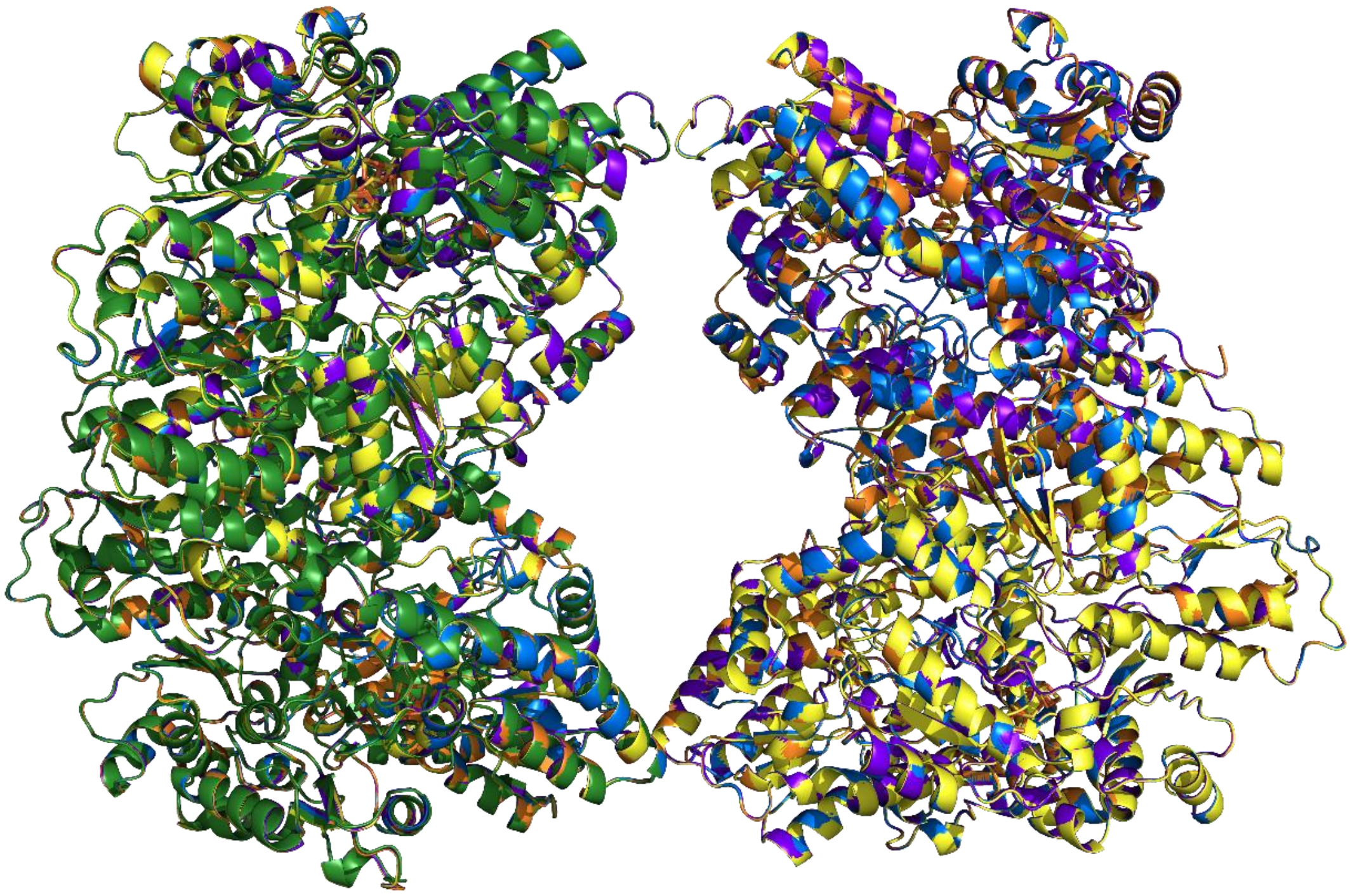

| Potential | +150 mV | +50 mV | -150 mV | -200 mV |
|---|---|---|---|---|
| RMSD (Å) | 0.23 | 0.07 | 0.07 | 0.10 |

**Figure S13**. Superposition of MoFe structures poised at each redox potential with Root-Mean-Square-Deviations (RMSD) of Cα positions, calculated in COOT, relative to methyl viologen poised at -400 mV. Structures are colored as follows: green (phenazine methosulfate poised at +150 mV), blue (methylene blue poised at +50 mV), orange (flavin mononucleotide poised at -150 mV), yellow (phenosafranin poised at -200 mV) and purple (methyl viologen poised at -400 mV); potentials quoted vs SHE.

## S13. Comparing $P^{1+}$ state in MoFe and VFe proteins

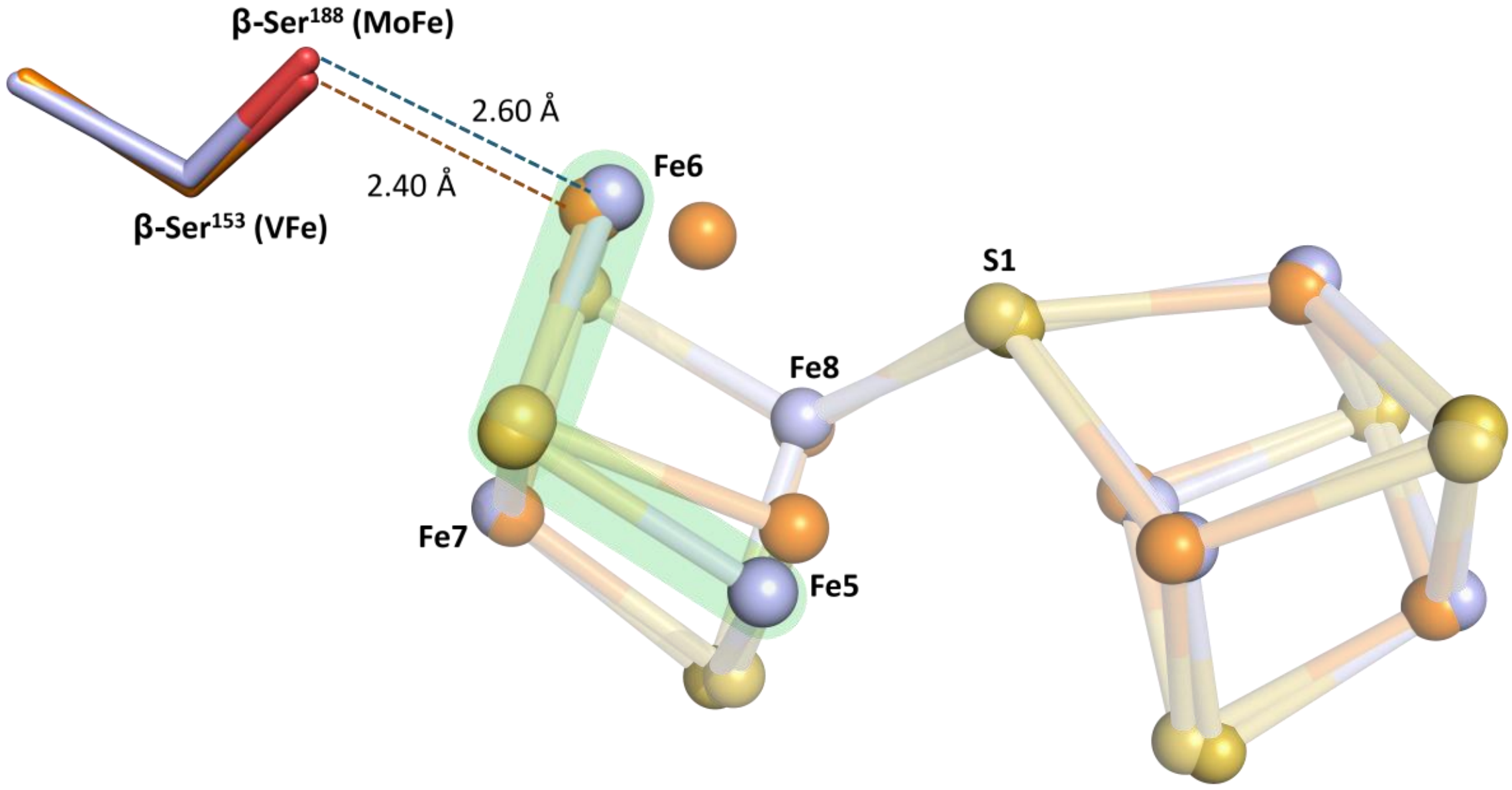


**Figure S14.** Structural overlay of $P^{1+}$ state isolated in MoFe protein in this work (PDB: 29UN; iron atom position shown in blue with the distal-half of the cluster highlighted in green), and VFe protein (PDB: 6FEA, iron atom positions shown in orange). VFe protein P-cluster was solved as a mixture of 2 states ($P^{1+}$, 40% and $P^{N}$, 60%).[24] Bond distance between **β-Ser$^{188}$—Fe6** in MoFe is 2.59 Å and for VFe it is **β-Ser$^{158}$—Fe6** = 2.40 Å.

**Table S12**. Selected averaged bond lengths and angles of $P^{1+}$ state in MoFe (this work, PDB: 29UN) and $P^{1+}$ in VFe (PDB: 6FEA).

| Bond Length (Å) | MoFe | Bond Length (Å) | VFe |
|---|---|---|---|
| β-Ser$^{188}$ – Fe6 | 2.59 | β-Ser$^{153}$ – Fe6 | 2.53 |
| S1 – Fe6 | 3.34 | S1 – Fe6 | 3.25 |
| α-Cys$^{153}$ – Fe6 | 2.35 | α-Cys$^{115}$ – Fe6 | 2.42 |
| S1 – Fe5 | 3.01 | S1 – Fe5 | 2.46 |
| α-Cys$^{88}$ – Fe5 | 2.19 | α-Cys$^{75}$ – Fe5 | 2.35 |
| α-Cys(N)$^{88}$ – Fe5 | 2.84 | α-Cys(N)$^{75}$ – Fe5 | 3.31 |
| β-Cys$^{95}$ – Fe8 | 2.35 | β-Cys$^{56}$ – Fe8 | 2.30 |
| β-Cys$^{70}$ – Fe7 | 2.35 | β-Cys$^{31}$ – Fe7 | 2.30 |
| α-Cys$^{88}$ – Fe4 | 2.31 | α-Cys$^{75}$ – Fe4 | 2.34 |
| α-Cys$^{62}$ – Fe3 | 2.32 | α-Cys$^{49}$ – Fe3 | 2.30 |
| β-Cys$^{95}$ – Fe2 | 2.28 | β-Cys$^{56}$ – Fe2 | 2.40 |
| β-C(N)$^{95}$ – Fe2 | 3.17 | β-Cys(N)$^{56}$ – Fe2 | 3.24 |
| α-Cys$^{154}$ – Fe1 | 2.39 | α-Cys$^{15}$ – Fe1 | 2.34 |

| Bond Angle (º) | MoFe | Bond Angle (º) | VFe |
|---|---|---|---|
| Fe6 – S2B – Fe5 | 91.81 | Fe6 – S2B – Fe5 | 83.88 |
| Fe6 – S1 – Fe5 | 62.37 | Fe6 – S1 – Fe5 | 61.37 |
| S2B – Fe6 – S1 | 95.28 | S2B – Fe6 – S1 | 93.50 |
| S2B – Fe5 – S1 | 106.09 | S2B – Fe5 – S1 | 115.92 |
| Fe5 – α-Cys$^{88}$ – Fe4 | 90.50 | Fe5 – α-Cys$^{75}$ – Fe4 | 75.91 |
| α-Cys(N)$^{88}$ – Fe5 – α-Cys$^{88}$ | 69.19 | α-Cys(N)$^{75}$ – Fe5 – α-Cys$^{88}$ | 65.60 |

### S14. $P^{3+}$ State Characterization

Perpendicular EPR measurements revealed a broad feature at $g_{eff}$ = 14 that derives, $g_{eff} \sim 2g\langle\hat{S}_z\rangle$, from the ground spin state of mixed character, for example $|\psi_\pm\rangle$ = -0.9998|-7/2⟩ + 0.01982|-3/2⟩ - 5.54x10$^{-4}$|1/2⟩ +… and $|\psi_-\rangle$ = +0.9998|7/2⟩ - 0.01982|3/2⟩ + 5.54x10$^{-4}$|-1/2⟩ +…, by rhombic mixing terms as described in the complete spin Hamiltonian matrix.[25]

**S14.1 Variable Temperature Parallel Mode EPR Spectra**

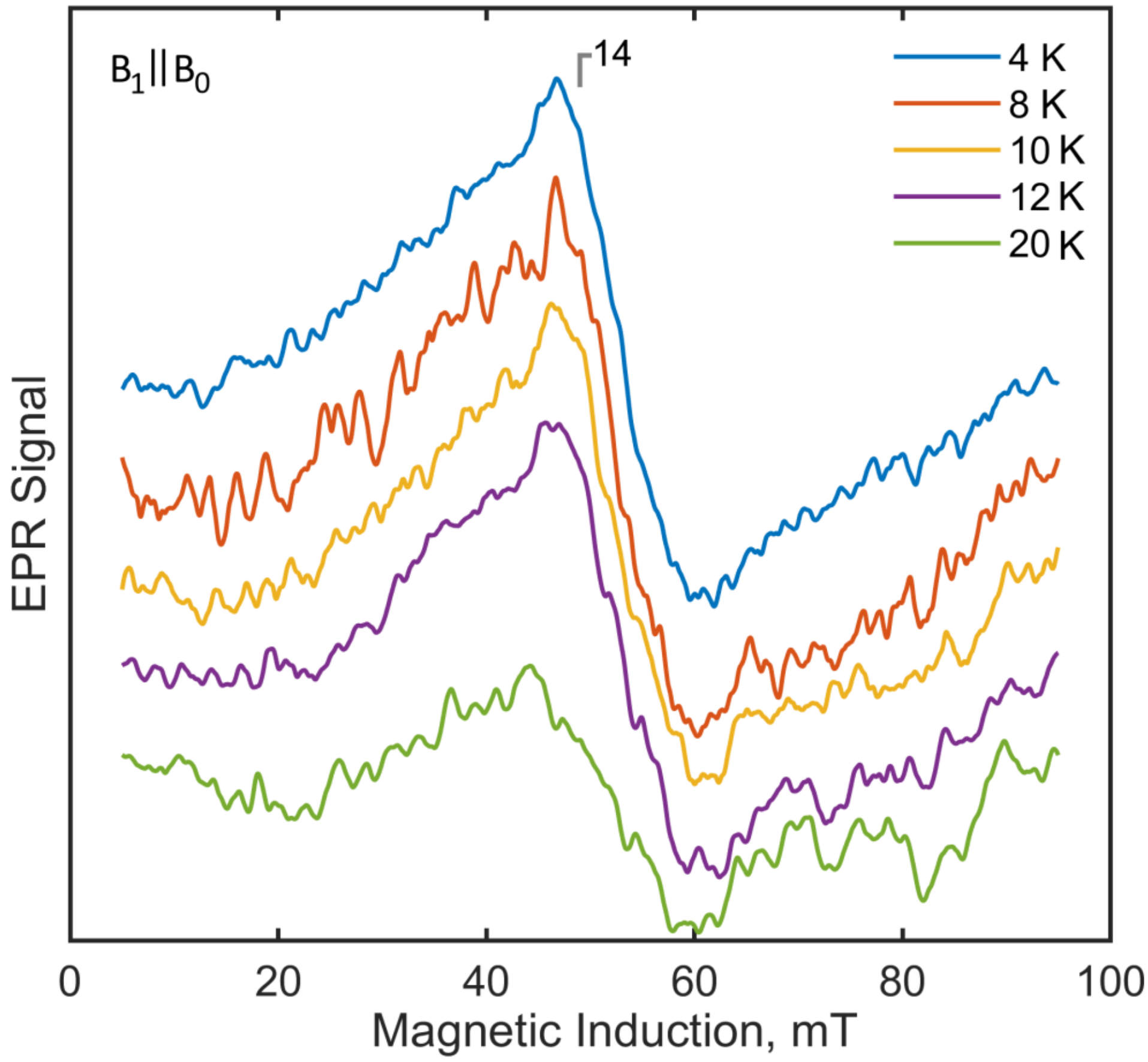


**Figure S15.** Variable temperature parallel mode X-band CW-EPR of MoFe microcrystals at +150 mV of $g$ = 14. EPR was collected at 9.390 GHz and 0.5 mT field modulation with microwave powers of (1.6,1.6, 25.2, 25.2, 100 mW) for (4, 8, 10, 12, 20 K), respectively using the ER4116DM dual mode resonator.

## S14.2 Variable Temperature Perpendicular Mode EPR Spectra

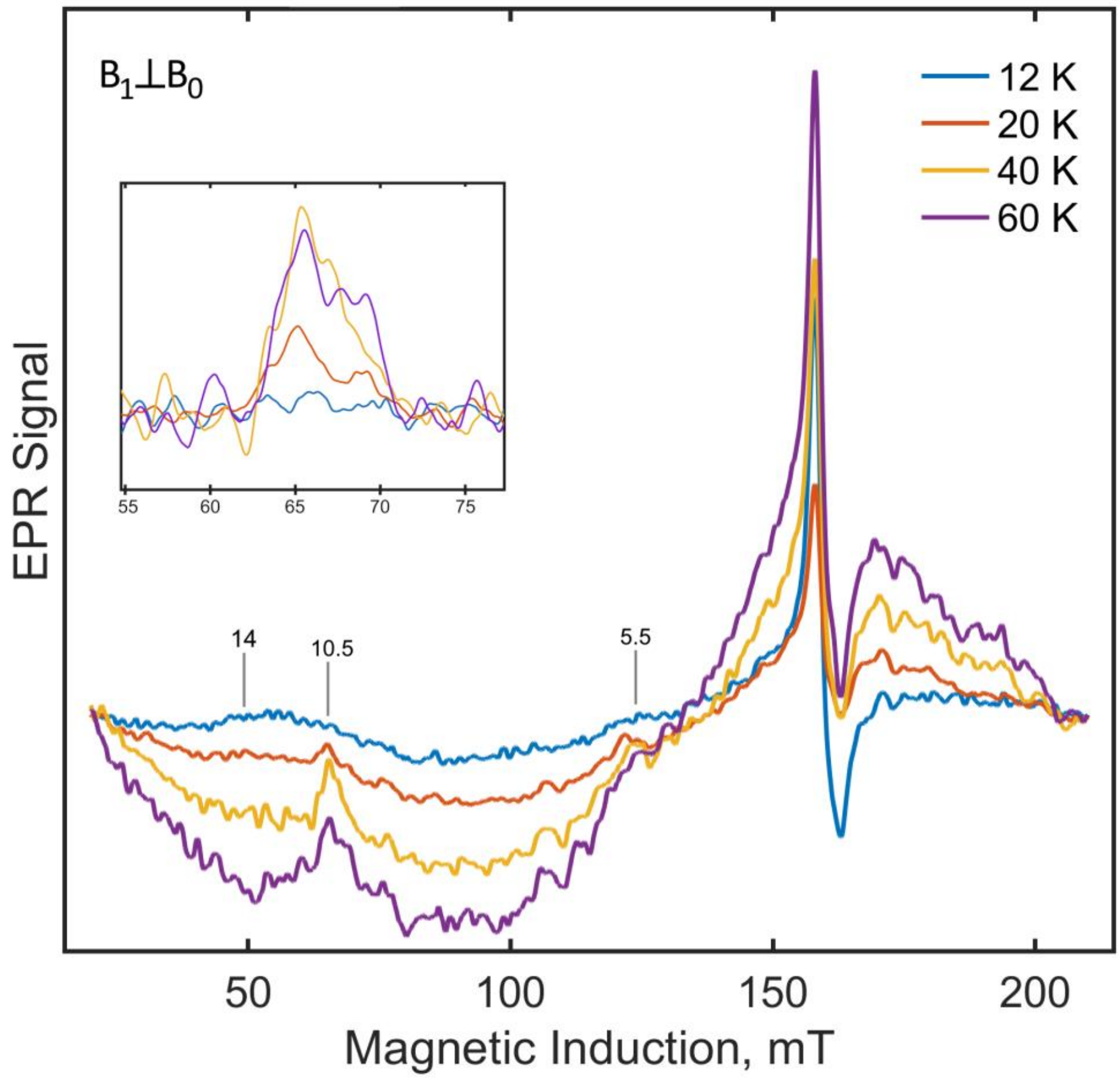


**Figure S16.** Variable temperature perpendicular mode X-band CW-EPR of MoFe microcrystals at +150 mV. Insert is an expansion of the region for *g* = 10.5 that has been baseline corrected. EPR spectra were collected at 9.645 GHz and 0.5 mT field modulation with microwave powers of 10 mW for 12 K and others at 200 mW, using the ER4116DM dual mode resonator.

## S14.3 Thermal Population

Variable temperature EPR shows the decrease of the $g_{eff}$ = 14 peak in both detection modes and the increase of the $g$ = 10.5 peak as a result of thermal population of |±5/2⟩ consistent with prior determination of zero field splitting of D = -3.7 $cm^{-1}$ and E/D=0.043.[25]

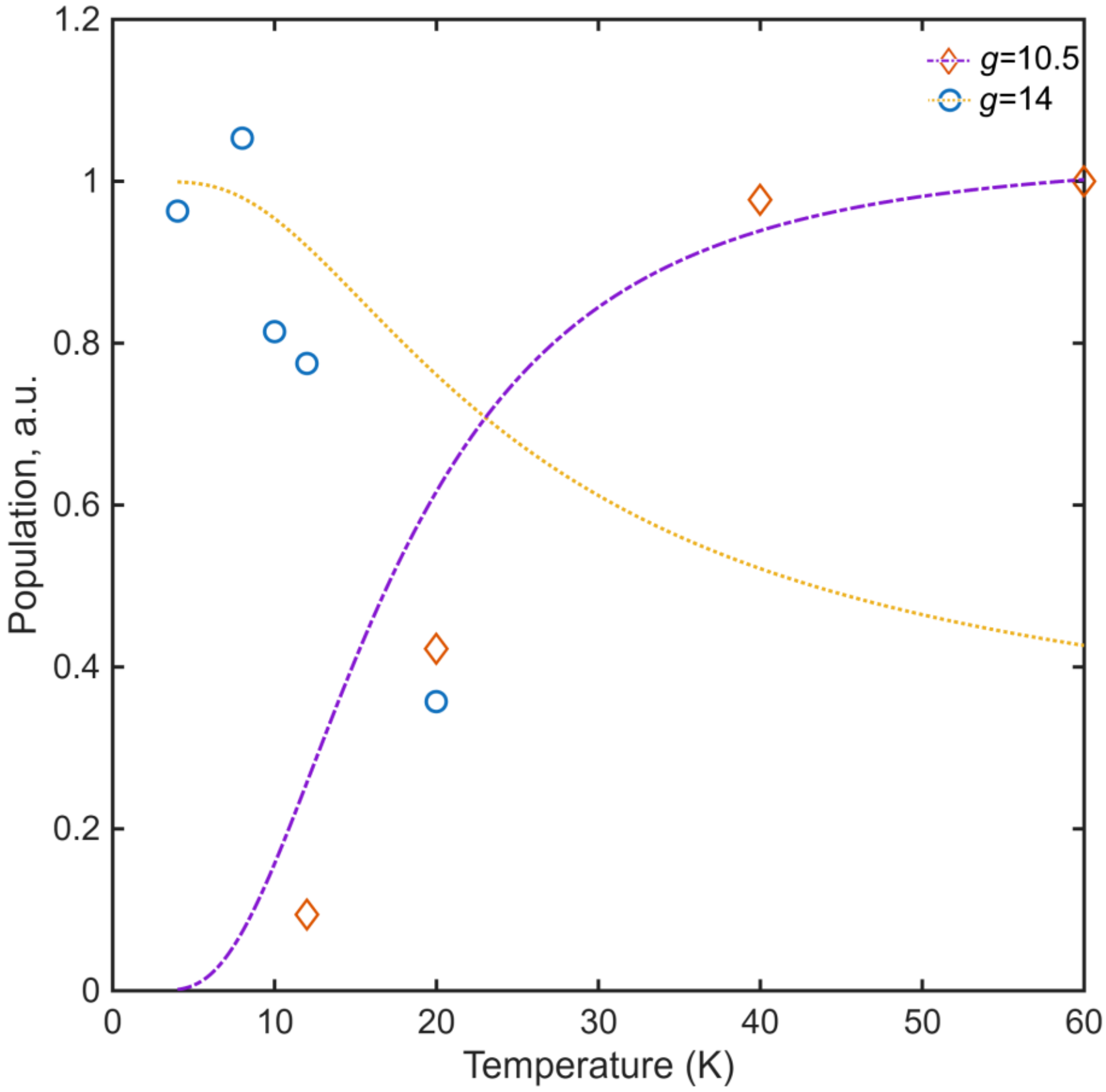


**Figure S17.** Calculation of thermal depopulation of ground state effective doublet of $g$ = 14 (yellow line) with respect to the 6D separation to the higher "|±5/2⟩" effective spin doublet state, $g$ = 10.5 and population of the higher state (purple line) scaled arbitrarily. Population intensities were derived from integrals of peaks from Figure S15-S16.

## S14.4 Comparing $P^{3+}$ and $P^{2+}$ state in MoFe protein

**Table S13**. Selected bond lengths and angles of the P-cluster and first coordination sphere for Chain A at +150 mV and +50 mV.

| **Bond Length (Å)** | **+150mV** | **+50 mV** |
|---|---|---|
| Species | $P^{3+}$ | $P^{2+}$ |
| β-$Ser^{188}$ – Fe6 | 1.91 | 1.99 |
| S1 – Fe6 | 3.69 | 3.84 |
| α-$Cys^{153}$ – Fe6 | 2.14 | 2.36 |
| S1 – Fe5 | 3.52 | 3.90 |
| α-$Cys^{88}$ – Fe5 | 2.29 | 2.28 |
| α-$Cys(N)^{88}$ – Fe5 | 2.42 | 2.28 |
| β-$Cys^{95}$ – Fe8 | 2.49 | 2.35 |
| β-$Cys^{70}$ – Fe7 | 2.42 | 2.39 |
| α-$Cys^{88}$ – Fe4 | 2.42 | 2.24 |
| α-$Cys^{62}$ – Fe3 | 2.19 | 2.27 |
| β-$Cys^{95}$ – Fe2 | 2.45 | 2.32 |
| β-$C(N)^{95}$ – Fe2 | 3.27 | 3.24 |
| α-$Cys^{154}$ – Fe1 | 2.29 | 2.20 |

| **Bond Angle (º)** | | |
|---|---|---|
| Fe6 – S2B – Fe5 | 121.64 | 114.43 |
| Fe6 – S1 – Fe5 | 67.76 | 56.04 |
| S2B – Fe6 – S1 | 79.76 | 88.01 |
| S2B – Fe5 – S1 | 84.30 | 91.53 |
| Fe5 – α-$Cys^{88}$ – Fe4 | 96.75 | 105.88 |
| α-$Cys(N)^{88}$ – Fe5 – α-$Cys^{88}$ | 78.31 | 82.23 |

## S14.5 Electron Density (2mFo-DFc) maps around P-cluster

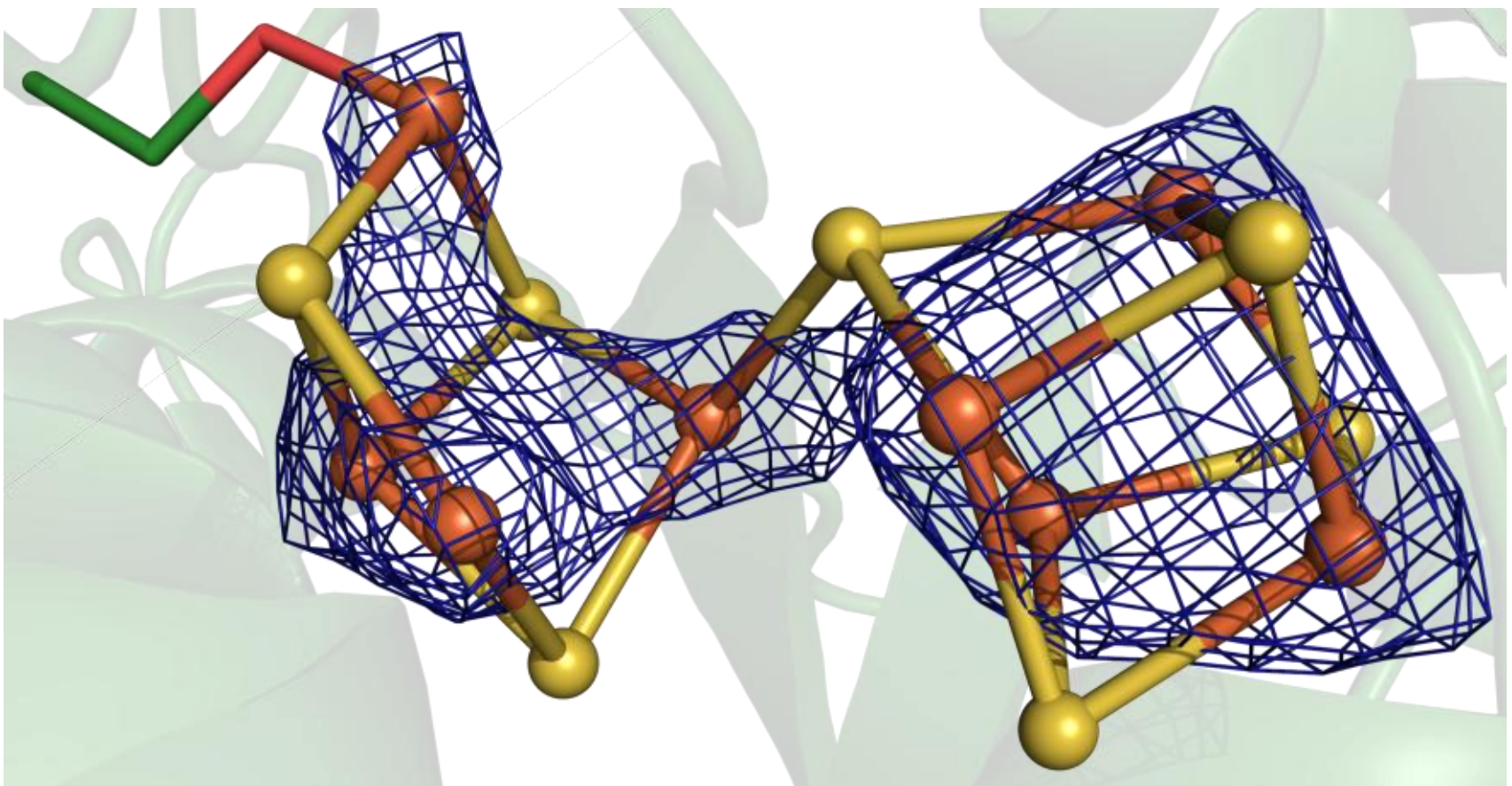

**Figure S18.** Maps of poised structures 2mFo-DFc maps (blue mesh) around the P-cluster contoured to 5σ in PyMOL for +150 mV poised structure of MoFe protein.

## S15. References


(1) Christiansen, J.; Goodwin, P. J.; Lanzilotta, W. N.; Seefeldt, L. C.; Dean, D. R. Catalytic and Biophysical Properties of a Nitrogenase Apo-MoFe Protein Produced by a nifB-Deletion Mutant of Azotobacter vinelandii. *Biochemistry* **1998**, *37* (36), 12611–12623. DOI: 10.1021/bi981165b.

(2) Jiménez-Vicente, E.; Martin Del Campo, J. S.; Yang, Z.-Y.; Cash, V. L.; Dean, D. R.; Seefeldt, L. C. Chapter Nine - Application of affinity purification methods for analysis of the nitrogenase system from Azotobacter vinelandii. In *Methods in Enzymology*, Armstrong, F. Ed.; Vol. 613; Academic Press, 2018; pp 231–255.

(3) Allen J. Bard, L. R. F. *Electrochemical Methods: Fundamentals and Applications*; Wiley, 2001.

(4) Laxmi, S.; Jaho, S.; Myers, W. K.; Vincent, K. A.; Carr, S. B. Crystallise, poise, capture: a multimodal platform for correlated structural and spectroscopic characterisation of redox enzymes. *JBIC Journal of Biological Inorganic Chemistry* **2026**. DOI: 10.1007/s00775-026-02148-x.

(5) Winter, G.; Waterman, D. G.; Parkhurst, J. M.; Brewster, A. S.; Gildea, R. J.; Gerstel, M.; Fuentes-Montero, L.; Vollmar, M.; Michels-Clark, T.; Young, I. D.; et al. DIALS: implementation and evaluation of a new integration package. *Acta Crystallographica Section D* **2018**, *74* (2), 85–97. DOI: doi:10.1107/S2059798317017235.

(6) Evans, P. R.; Murshudov, G. N. How good are my data and what is the resolution? *Acta Crystallographica Section D* **2013**, *69* (7), 1204–1214. DOI: doi:10.1107/S0907444913000061.

(7) Winter, G. xia2: an expert system for macromolecular crystallography data reduction. *Journal of Applied Crystallography* **2010**, *43* (1), 186–190. DOI: doi:10.1107/S0021889809045701.

*(8) McCoy, A. J.; Grosse-Kunstleve, R. W.; Adams, P. D.; Winn, M. D.; Storoni, L. C.; Read, R. J. Phaser crystallographic software. Journal of Applied Crystallography* ***2007****, 40 (4), 658–674. DOI: doi:10.1107/S0021889807021206.*

(9) Emsley, P.; Lohkamp, B.; Scott, W. G.; Cowtan, K. Features and development of Coot. *Acta Crystallographica Section D* **2010**, *66* (4), 486–501. DOI: doi:10.1107/S0907444910007493.

(10) Murshudov, G. N.; Skubak, P.; Lebedev, A. A.; Pannu, N. S.; Steiner, R. A.; Nicholls, R. A.; Winn, M. D.; Long, F.; Vagin, A. A. REFMAC5 for the refinement of macromolecular crystal structures. *Acta Crystallographica Section D* **2011**, *67* (4), 355–367. DOI: doi:10.1107/S0907444911001314.

(11) Afonine, P. V.; Mustyakimov, M.; Grosse-Kunstleve, R. W.; Moriarty, N. W.; Langan, P.; Adams, P. D. Joint X-ray and neutron refinement with phenix.refine. *Acta Crystallographica Section D* **2010**, *66* (11), 1153–1163. DOI: doi:10.1107/S0907444910026582.

(12) Schrodinger, LLC. The PyMOL Molecular Graphics System, Version 1.8. 2015. Schrodinger, LLC. The JyMOL Molecular Graphics Development Component, Version 1.8. 2015. Schrodinger, LLC. The AxPyMOL Molecular Graphics Plugin for Microsoft PowerPoint, Version 1.8. 2015.

(13) Richardson, J. S.; Williams, C. J.; Hintze, B. J.; Chen, V. B.; Prisant, M. G.; Videau, L. L.; Richardson, D. C. Model validation: local diagnosis, correction and when to quit. *Acta Crystallographica Section D* **2018**, *74* (2), 132–142. DOI: doi:10.1107/S2059798317009834.

(14) Lanzilotta, W. N.; Christiansen, J.; Dean, D. R.; Seefeldt, L. C. Evidence for Coupled Electron and Proton Transfer in the [8Fe-7S] Cluster of Nitrogenase. *Biochemistry* **1998**, *37* (32), 11376–11384. DOI: 10.1021/bi980048d.

(15) Pierik, A. J.; Wassink, H.; Haaker, H.; Hagen, W. R. Redox properties and EPR spectroscopy of the P clusters of Azotobacter vinelandii MoFe protein. *European Journal of Biochemistry* **1993**, *212* (1), 51–61. DOI: https://doi.org/10.1111/j.1432-1033.1993.tb17632.x.

(16) Juers, D. H.; Ruffin, J. MAP_CHANNELS: a computation tool to aid in the visualization and characterization of solvent channels in macromolecular crystals. *Journal of Applied Crystallography* **2014**, *47* (6), 2105–2108. DOI: doi:10.1107/S160057671402281X.

(17) Ash, P. A.; Kendall-Price, S. E. T.; Evans, R. M.; Carr, S. B.; Brasnett, A. R.; Morra, S.; Rowbotham, J. S.; Hidalgo, R.; Healy, A. J.; Cinque, G.; et al. The crystalline state as a dynamic system: IR microspectroscopy under electrochemical control for a [NiFe] hydrogenase. *Chemical Science* **2021**, *12* (39), 12959–12970, 10.1039/D1SC01734A. DOI: 10.1039/D1SC01734A.

(18) Zhan, R.; Song, S.; Liu, Y.; Dong, S. Mechanisms of methylene blue electrode processes studied by in situ electron paramagnetic resonance and ultraviolet–visible spectroelectrochemistry. *Journal of the Chemical Society, Faraday Transactions* **1990**, *86* (18), 3125–3127, 10.1039/FT9908603125. DOI: 10.1039/FT9908603125.

(19) Herder, J. A.; Kruse, S. J.; Nicholas, A. D.; Forbes, T. Z.; Walter, E. D.; Cho, H.; Cahill, C. L. Systematic Study of Solid-State U(VI) Photoreactivity: Long-Lived Radicalization and Electron Transfer in Uranyl Tetrachloride. *Inorganic Chemistry* **2024**, *63* (11), 4957–4971. DOI: 10.1021/acs.inorgchem.3c04144.

(20) Dahl, P. J.; Pellows, L. M.; Yang, Z.-Y.; Seefeldt, L. C.; Peters, J. W.; Dukovic, G.; Mulder, D. W.; King, P. W. Pre-steady-state kinetics of nanocrystal:molybdenum nitrogenase biohybrids reveals hole-scavenging efficiency is critical to N<sub>2</sub> reduction. *Cell Reports Physical Science* **2025**, *6* (8). DOI: 10.1016/j.xcrp.2025.102732.

(21) Stoll, S.; Schweiger, A. EasySpin, a comprehensive software package for spectral simulation and analysis in EPR. *Journal of Magnetic Resonance* **2006**, *178* (1), 42–55. DOI: https://doi.org/10.1016/j.jmr.2005.08.013.

(22) Cao, L.; Börner, M. C.; Bergmann, J.; Caldararu, O.; Ryde, U. Geometry and Electronic Structure of the P-Cluster in Nitrogenase Studied by Combined Quantum Mechanical and Molecular Mechanical Calculations and Quantum Refinement. *Inorganic Chemistry* **2019**, *58* (15), 9672–9690. DOI: 10.1021/acs.inorgchem.9b00400.

(23) Maslać, N.; Törer, M. R.; Bolte, P.; Wagner, T. Molecular basis of N<sub>2</sub> fixation in a hyperthermophilic archaeon. *bioRxiv* **2025**, 2025.2010.2010.681579. DOI: 10.1101/2025.10.10.681579.

(24) Sippel, D.; Rohde, M.; Netzer, J.; Trncik, C.; Gies, J.; Grunau, K.; Djurdjevic, I.; Decamps, L.; Andrade, S. L. A.; Einsle, O. A bound reaction intermediate sheds light on the mechanism of nitrogenase. *Science* **2018**, *359* (6383), 1484–1489. DOI: 10.1126/science.aar2765.

(25) Hagen, W. R.; Wassink, H.; Eady, R. R.; Smith, B. E.; Haaker, H. Quantitative EPR of an S= 7/2 system in thionine-oxidized MoFe proteins of nitrogenase. *European Journal of Biochemistry* **1987**, *169* (3), 457–465. DOI: https://doi.org/10.1111/j.1432-1033.1987.tb13633.x.
